# Placental Mesenchymal Stem Cells Promote the Polarization of Astrocytes Towards the A2 Phenotype by Modulating the TGF-β1 Signaling Pathway in Cerebral Ischemia–Reperfusion Rats

**DOI:** 10.3390/biom16081103

**Published:** 2026-07-28

**Authors:** Ningmei Liu, Yanyan Fu, Taojuan Wu, Dongmei Chen, Ting Liu, Haibin Ma, Chuanshang Cao, Binwu Ma, Jianguo Niu, Xueyun Liang

**Affiliations:** 1 Key Laboratory of Ningxia Stem Cell and Regenerative Medicine, Institute of Medical Sciences, General Hospital of Ningxia Medical University, Yinchuan 750001, China; ningmeizxyy@163.com (N.L.); fyy292505@163.com (Y.F.); wtj3117001570@163.com (T.W.); chendm1981@163.com (D.C.); tingliu512@163.com (T.L.); dreamcell@163.com (H.M.); c2445201140@163.com (C.C.); 2 Neurology Department, General Hospital of Ningxia Medical University, Yinchuan 750001, China; nxmbw@126.com; 3 Ningxia Key Laboratory of Cerebrocranial Diseases, School of Basic Medical Sciences, Ningxia Medical University, Yinchuan 750004, China; niujg@nxmu.edu.cn

**Keywords:** mesenchymal stem cells, astrocytes, polarization, TGF-β1 signaling pathway, cerebral ischemia–reperfusion

## Abstract

Astrocytic function imbalance is a key factor affecting cerebral ischemia rehabilitation. Mesenchymal stem cells have therapeutic potential for cerebral ischemia, but their mechanisms remain unclear. This study explored how placental mesenchymal stem cells pro-mote astrocytes towards the A2 phenotype, which benefits the repair of cerebral ischemia injury. We established the animal model of middle cerebral artery ischemia/reperfusion and an in vitro interleukin-1β-treated astrocyte inflammation model and utilized neuro-logical function assessment, 2,3,5-triphenyltetrazolium chloride staining, immunofluorescence staining, Western blotting and other methods to evaluate the impact and associated mechanisms of transplanting placental mesenchymal stem cells into rats with cerebral ischemia–reperfusion injuries. The results demonstrated that transplantation of placental mesenchymal stem cells ameliorated neurological dysfunction and histopathological damage, alleviated neuroinflammation and enhanced neurotrophic factor levels, promoted the polarization of interleukin-1β-treated astrocytes toward the A2 phenotype, and suppressed the abundance of core mediators within the cerebral TGF β1/Smad signaling cascade in ischemia/reperfusion model. Collectively, placental mesenchymal stem cell grafting facilitates the polarization of cerebral astrocytes toward the protective A2 phenotype, presumably by regulating the TGF β1/Smad signaling cascade in ischemic brain injury.

## 1. Introduction

Ischemic stroke represents a severe global public health challenge lacking effective therapeutic interventions and is a highly harmful illness characterized by soaring incidence and elevated mortality rate [1]. Many ischemic stroke patients present with varying degrees of neurological dysfunction and other sequelae [2]. Cerebral ischemia–reperfusion injury (CIRI) serves as a core pathological mechanism underlying ischemic stroke deterioration [3], typically occurring after reperfusion therapies like thrombolysis or mechanical thrombectomy. Neuroinflammation is a major contributor to CIRI and underlies prolonged neurological dysfunction [4].

Astrocytes execute essential modulatory effects in CIRI-associated neuroinflammation, and combined with other cells, contribute to the neuroinflammatory response following the CIRI cascade [5]. Following CIRI, quiescent astrocytes undergo reactive activation and mainly differentiate into two distinct functional subtypes, namely the pro-inflammatory A1 and neuroprotective A2 phenotypes, and when excessively activated, astrocytes can adopt a neurotoxic A1 profile, releasing a broad array of inflammatory mediators that exacerbate neuroinflammation [6]. Astrocytes thus represent one of the key target cells in the therapy of CIRI [7,8].

The transforming growth factor-β (TGF-β) cascade exerts essential modulatory effects on a wide spectrum of biological events, encompassing immune regulation, cell proliferation, survival, senescence and tissue repair [9]. Accumulating mechanistic evidence has validated the functional involvement of the TGF-β1/Smad signaling cascade in astrocyte activation [10] and the production of glial fibrillary acidic protein (GFAP), a major active marker of astrocytes [11]. Thus, we hypothesize that modulating the TGF-β signaling pathway in astrocytes may contribute to the regulation of astrocyte activation.

Despite the availability of multiple treatment strategies for ischemic brain injury, such as thrombolytic therapy, antiplatelet therapy and neuroprotective agents, their efficacy remains limited [12]. Mesenchymal stem cell (MSC) transplantation is an emerging therapeutic strategy for neurological injury with notable therapeutic advantages [13]. Prior investigations conducted by our team verify that placental mesenchymal stromal cells (PMSCs) possess substantial potential for the treatment of neuroinflammation [14]. However, whether PMSCs confer their anti-inflammatory effects by modulating the astrocytic TGF-β1 signaling pathway remains poorly defined and warrants further investigation.

This preclinical investigation sought to characterize whether PMSC delivery could suppress neuroinflammatory cascades through tuning TGF-β1 signaling, a molecular axis governing glial functional remodeling, to trigger the conversion of harmful A1 reactive astrocytes toward a neuroprotective A2 subtype. The experimental data presented herein delineate the mechanistic foundation underlying PMSC therapeutic capacity, and provide preclinical proof for bench-to-bedside translation of a PMSC-based regimen for designing subsequent early-stage clinical studies targeting CIRI.

## 2. Materials and Methods

### 2.1. Animals

A total of 52 mature male Sprague–Dawley rats with body weights ranging from 250 to 280 g were sourced from institutional experimental animal facilities of Ningxia Medical University. Three distinct experimental groups were generated via random allocation of laboratory rats: Sham (*n* = 16), MCAO/R (*n* = 20), and PMSC (*n* = 16). All rat subjects were sustained in standardized barrier housing with constant ambient temperature set between 22 and 25 °C, steady relative humidity at 55%, and housing cycles followed a 12 h light–dark shift regime, with bright illumination supplied between 8 a.m. and 8 p.m. Standard chow and sterile drinking water were accessible to all animals ad libitum throughout the whole experimental observation period. Formal ethical authorization, issued by the Ethics Committee of the General Hospital of Ningxia Medical University (Reference No. KYLL-2021-581), was obtained for all in vivo manipulation schemes and experimental workflows described in this investigation. All animal-related operations were implemented in strict accordance with the internationally recognized Guidelines for the Care and Use of Laboratory Animals.

### 2.2. Cell Culture

All PMSCs at the fifth passage (P5) utilized for experimental analyses in this work were supplied by the Ningxia Key Laboratory of Stem Cell and Regenerative Medicine. Cell cultivation was performed using serum-free MSC Basal medium (Yinfeng, China) in constant-temperature culture chambers (37 °C) containing 5% carbon dioxide under humid conditions. Derived from human placental tissue specimens, these PMSCs displayed consistent MSC phenotypic characteristics, with positive expression of canonical surface antigens, including CD73, CD90 and CD105, and absent expression of CD14, CD45, and CD34, as well as HLA-DR, consistent with results reported in earlier literature [15]. All operational protocols related to cell harvesting and specimen collection were authorized by the Ethics Committee of the General Hospital of Ningxia Medical University (Ethics Approval No. 2020-289).

Cortical astrocytes used for primary cultivation were isolated from cerebral specimens of neonatal rats within postnatal days 1 to 3, adopting standardized glial cell preparation protocols. After surgical collection of cerebral cortical specimens, enzymatic digestion was conducted by incubation with TrypLE^TM^ reagent at a constant temperature of 37 °C for 15 min. Post-digestion cortical cellular components were resuspended and cultivated in complete Dulbecco’s Modified Eagle Medium with 10% fetal bovine serum in constant-temperature culture chambers (37 °C) containing 5% carbon dioxide under humid conditions. Culture medium was replaced every three days throughout the entire cultivation period. The phenotypic identity and purity of cultured astrocytes were characterized through GFAP immunofluorescence labeling. Only cellular cultures exhibiting GFAP-positive staining proportions exceeding 80% were utilized for subsequent experimental validation and functional analyses.

For induction of an in vitro inflammatory model, astrocytes at 80% confluency were stimulated with IL-1β (100 ng/mL) for 24 h.

### 2.3. Middle Cerebral Artery Occlusion–Reperfusion (MCAO/R) Model Establishment

General anesthesia was induced in all experimental rats via intramuscular co-administration of xylazine (40 mg/kg) and Zoletil 50 (50 mg/kg). A heating pad was utilized throughout the entire surgical procedure to maintain the rectal temperature of each rodent at 37 ± 0.5 °C. Following fur trimming and aseptic decontamination of the cervical operative field, a 1–3 cm midline cervical incision was made to fully unveil the left carotid arterial system, followed by permanent occlusion of the external carotid branch, and a customized filament occlude featuring a 0.2 mm main body and a 0.36 mm silicone-coated tip was carefully inserted into the internal carotid artery to establish MCAO lasting 90 min. When the occlusion period ended, the embolus was retracted, and the insertion site of the arterial segment was clamped to prevent retrograde hemorrhage.

All animals were transferred onto a heating pad maintained at 37 °C postoperatively and monitored uninterruptedly until complete recovery from anesthesia. Subsequently, rats were housed individually, with persistent surveillance of vital physiological parameters to facilitate uncomplicated postoperative recovery. Animals allocated to the Sham group underwent identical surgical dissection without filament-mediated MCAO. For perioperative analgesia, carprofen was administered with 5 mg/kg prior to the return of consciousness, with repeated injections scheduled every 24 h for a total of 72 postoperative hours. Daily physical assessments were carried out to evaluate manifestations of pain, general distress, and the healing progression of surgical wounds.

### 2.4. PMSC Transplantation

Following MCAO/R, on the same day, the conscious rats were evaluated using the Zea Longa scoring system to assess the success of the model; animals given a score of 1–2 were included in the study. Upon confirmation, anesthesia was maintained using isoflurane, and the hair on the rats’ heads was shaved. The rats were then securely positioned in a stereotaxic frame with the skull aligned horizontally. A scalp incision was made to expose the bregma, and a 1 mm burr hole was drilled at coordinates 0.24 mm anterior and 4.0 mm lateral to the bregma. After puncturing the dura, the needle was carefully advanced 3.5 mm along the Z-axis, and 30 μL of a suspension containing 1 × 10^6^ PMSCs with approximately 100% cell viability was injected at a controlled rate to ensure gradual cell delivery. The needle was held in place for 10 min to allow for complete cell dispersion before being withdrawn slowly to prevent a sudden increase in intracranial pressure. The Sham-operated rats were injected with 30 μL PBS.

### 2.5. Neurological Function Assessment

Neurological deficits in rats were evaluated using the Zea Longa scoring system, which ranges from 0 to 4 points, with higher scores meaning more severe neurological impairment due to inflammation. The scores indicate the following: 0: No neurological deficit. 1: Mild deficit: the contralateral (usually left) forelimb cannot be fully extended when the rat is lifted by the tail; forelimb flexion is present. 2: Moderate deficit: the animal circles toward the ischemic (contralateral) side while walking. 3: Severe deficit: the animal falls or topples toward the ischemic side when standing or crawling. 4: Very severe deficit: no spontaneous walking and/or loss of consciousness.

Additionally, the modified Garcia scoring system was used to assess neurological function, evaluating parameters such as spontaneous activity, posture, forelimb extension, grasping and climbing ability, bilateral body sensation and bilateral whisker response. The total score is 18, with lower scores indicating more severe neurological deficits.

### 2.6. 2,3,5-Triphenyltetrazolium Chloride (TTC) Staining

Upon finishing the 72 h reperfusion phase, all rats were humanely sacrificed for whole-brain tissue extraction, followed by immediate snap freezing at −80 °C for 3 min. A 2% TTC staining solution prepared with phosphate-buffered saline was freshly formulated during tissue freezing. Frozen brain specimens were sectioned coronally into 2 mm thick slices and incubated in the TTC working solution at 37 °C for 10–15 min; slices were flipped every 5 min to achieve uniform staining. All stained tissue sections were immersed in 4% paraformaldehyde (PFA) for 1 h for tissue fixation. Viable cerebral parenchyma exhibited bright red staining, while ischemic infarct lesions appeared pale white. ImageJ software version 1.53 was utilized to perform quantitative morphometric analysis of infarct areas. The edema-corrected infarct volume was calculated by the following formula: Veci = Vin × (1 − (Vip − Vcon))/Vcon. In this formula, Veci represents edema-corrected infarct volume, Vin indicates raw infarct volume, Vip refers to the volume of the ipsilateral cerebral hemisphere, and Vcon stands for the volume of the contralateral cerebral hemisphere.

### 2.7. Sample Preparation for Histological Analysis

After 72 h of reperfusion, transcranial perfusion was performed on the rats under deep anesthesia following heparinization to achieve rapid blood clearance and deliver fixative while preserving ultrastructure and antigenicity. After thoracotomy, the left ventricle was cannulated through the ascending aorta, and a large right atrial outlet was opened to establish drainage. The circuit was perfused with ice-cold PBS; when the liver cleared and effluent ran clear, the perfusate was switched to 4% PFA in PBS, pH ~7.4. Fixation endpoints included liver clearing, fixation tremors and limb rigidity. Isolated rat brain tissues were processed for hematoxylin and eosin (HE) staining, Nissl staining, and immunofluorescence staining. For frozen section preparation, specimens were post-fixed in 4% PFA at 4 °C for 24 h. Tissues were then cryoprotected in 30% sucrose–PBS solution until complete sedimentation of the tissue blocks. Following embedding in optimal cutting temperature medium, the samples were trimmed into 2 mm thick tissue blocks, snap-frozen in liquid nitrogen, and stored at −80 °C. Serial 8 μm thick frozen sections were subsequently sliced using a cryostat for subsequent histological staining.

### 2.8. Immunofluorescent Staining

Cellular and tissue phenotypic characteristics were determined by immunofluorescence staining. All tissue sections and cell coverslips underwent antigen retrieval in pH 6.0 citrate buffer, followed by 1 h of non-specific blocking with 5% BSA. Samples were subsequently incubated overnight at 4 °C with primary antibodies targeting GFAP (1:1000; Abcam, Cambridge, UK, ab4648), iNOS (1:500; Abcam, ab210823), and Arg-1 (1:800; Proteintech, Rosemont, IL, USA, 66129-1-Ig). After adequate rinsing, specimens were incubated with Alexa Fluor^®^ 488 or Alexa Fluor^®^ 555-conjugated secondary antibodies for 3 h in darkness, and DAPI staining was finally applied for nuclear counterstaining. Fluorescent images were captured via microscopy, and positive cells were quantified with ImageJ version 1.53 by a blinded investigator. Negative controls without primary antibodies showed no specific signals, verifying staining specificity.

### 2.9. Western Blot Assessment

Protein samples were extracted from injured cerebral tissues and cultured cells for Western blot assessment. Uniform protein loading of 50 μg per lane was separated using 10% or 12% sodium dodecyl sulfate–polyacrylamide gel electrophoresis, followed by electrophoretic transfer onto polyvinylidene fluoride membranes. After 1 h of room-temperature blocking, membranes were subjected to overnight incubation at 4 °C with a panel of primary antibodies, including rabbit anti-GFAP (1:2000; Abcam, ab4648), rabbit anti-GDNF (1:1000; Abcam, ab108319), mouse anti-Arg-1 (1:20,000; Proteintech, 66129-1-Ig), rabbit anti-S100A10 (1:25,000; Abcam, ab76472), rabbit anti-TGFβ1 (1:1000; Abcam, ab215715), rabbit anti-TβRI (1:1500; Proteintech, 30117-1-AP), mouse anti-TGFβRII (1:20,000; Proteintech, 66636-1-Ig), rabbit anti-p-Smad2/3 (1:1000; Abclonal, AP0548), rabbit anti-C3 (1:2000; Abcam, ab200999), rabbit anti-IL-6 (1:1000; Abcam, ab259341), and mouse anti-β-actin (1:1000; Proteintech, 66009-1-Ig). After thorough washing with TBST for three cycles, membranes were incubated with horseradish peroxidase-conjugated goat anti-rabbit IgG (1:10,000; Proteintech, SA00001-2) or goat anti-mouse IgG (1:10,000; Proteintech, SA00001-1) for 1 h at room temperature. Immunoreactive protein bands were visualized using an enhanced chemiluminescence detection system. All experiments were conducted in triplicate to ensure consistent and reproducible outcomes. Band intensity was quantitatively determined via densitometric analysis using ImageJ software (version 1.53, National Institutes of Health, Bethesda, MD, USA). Specifically, band intensities were determined by measuring relative grayscale values, and β-actin served as the internal reference for data normalization.

### 2.10. Total RNA Isolation and Quantitative Real-Time Polymerase Chain Reaction (qRT-PCR) Analysis

For the analysis of gene expression profiles, total RNA was extracted from injured brain tissues and cultured cells using Trizol reagent. Isolated RNA was reverse-transcribed into cDNA with a commercial Vazyme kit (Wuhan, China), and qPCR amplification was subsequently performed using a thermal cycler instrument (Thermo Fisher Scientific Inc., Shanghai, China). GAPDH was applied as the internal reference gene for expression normalization. Relative gene expression variations compared with control samples were calculated using the 2^−ΔΔCt^) (QuantStudio™ Design and Analysis Desktop Software v1.6. comparative quantification method. The corresponding primer sequences are listed as follows:

IL-6-Rat-F → GCCTTCTTGGGACTGATGTTGTTG

IL-6-Rat-R → GTCTGTTGTGGGTGGTATCCTCTG

IL-1β-Rat-F → TCTCACAGCAGCATCTCGACAAG

IL-1β-Rat-R → CCACGGGCAAGACATAGGTAGC

TNF-α-Rat-F → GTCTGTGCCTCAGCCTCTTC

TNF-α-Rat-R → TGGAACTGATGAGAGGGAGC

iNOS-Rat-F → GGAAGAGACGCACAGGCAGAG

iNOS-Rat-R → CAGCAGGCACACGCAATGATG

Arg-1-Rat-F → GAGGAGGTGACTCGTACTGTGAAC

Arg-1-Rat-R → GGCTTATGATTACCTTCCCGTTTCG

BDNF-Rat-F → AAGGCACTGGAACTCGCAATG

BDNF-Rat-R → TTATGAACCGCCAGCCAATTCTC

NGF-Rat-F → CATCCACCCACCCAGTCTTCC

NGF-Rat-R → TCCGTGGCTGTGGTCTTATCTC

TGFβ1-Rat-F → TGGACCGCAACAACGCAATC

TGFβ1-Rat-R → CACTGCTTCCCGAATGTCTGAC

TβRI-Rat-F → CATTGCTGGTCCAGTCTGCTTC

TβRI-Rat-R → TGGTGAATGACAGTGCGGTTATG

TβRII-Rat-F → GACCTTCTTCATGTGCTCCTGTAAC

TβRII-Rat-R → GGACTGCTGGTGGTGTATTCTTC

Smad2-Rat-F → GTCGTCCATCTTGCCATTCACTC

Smad2-Rat-R → GTTCTCCACCACCTGCTCCTC

Smad3-Rat-F → ATGTCGTCCATCCTGCCCTTC

Smad3-Rat-R → CTCGCACCACTTCTCCTCCTG

### 2.11. Enzyme-Linked Immunosorbent Assay (ELISA)

Levels of TNF-α and IL-6 in cell culture supernatants were measured using commercial sandwich ELISA kits (JiangLai BioTec, Shanghai, China; JL10484, JL20268) according to the manufacturer’s protocols. Supernatant samples and calibrators were loaded in duplicate onto precoated 96-well plates and incubated sufficiently. After washing off unbound residues, plates were incubated with biotinylated antibodies against TNF-α and IL-6, followed by streptavidin-horseradish peroxidase incubation. Color development was initiated by tetramethylbenzidine substrate, and the reaction was terminated with stop solution. Absorbance was immediately detected using a microplate reader (PerkinElmer VICTOR Nivo, Waltham, MA, USA). Sample cytokine concentrations were calculated based on standard curves established with kit-provided recombinant proteins. All samples were tested in duplicate, and average values were used for subsequent statistical analysis.

### 2.12. Statistics

Blinded evaluation was implemented throughout data collection and outcome assessment in this study. Every experimental group was designed with a minimum of three independent biological replicates, while each individual specimen was examined in triplicate technical repeats to diminish analytical variability. Post-experimental data sorting and statistical computation were completed via GraphPad Prism 9.0 software (GraphPad Software, La Jolla, CA, USA). Distribution normality of all datasets was validated using the Shapiro–Wilk test, which confirmed normal data distribution for all measured indicators. Intergroup differences among multiple cohorts were analyzed using one-way ANOVA (GraphPad Prism 9.0). Where significant overall statistical divergence existed, Tukey’s HSD post hoc analysis was further adopted for pairwise intergroup comparison. All quantitative measurements were exhibited in the form of mean ± standard deviation. Statistical significance was determined with a two-tailed threshold of *p* < 0.05.

## 3. Results

### 3.1. Transplantation of PMSCs Ameliorates Neurological Dysfunction and Histopathological Damage in MCAO/R Rats

**Figure 1:**
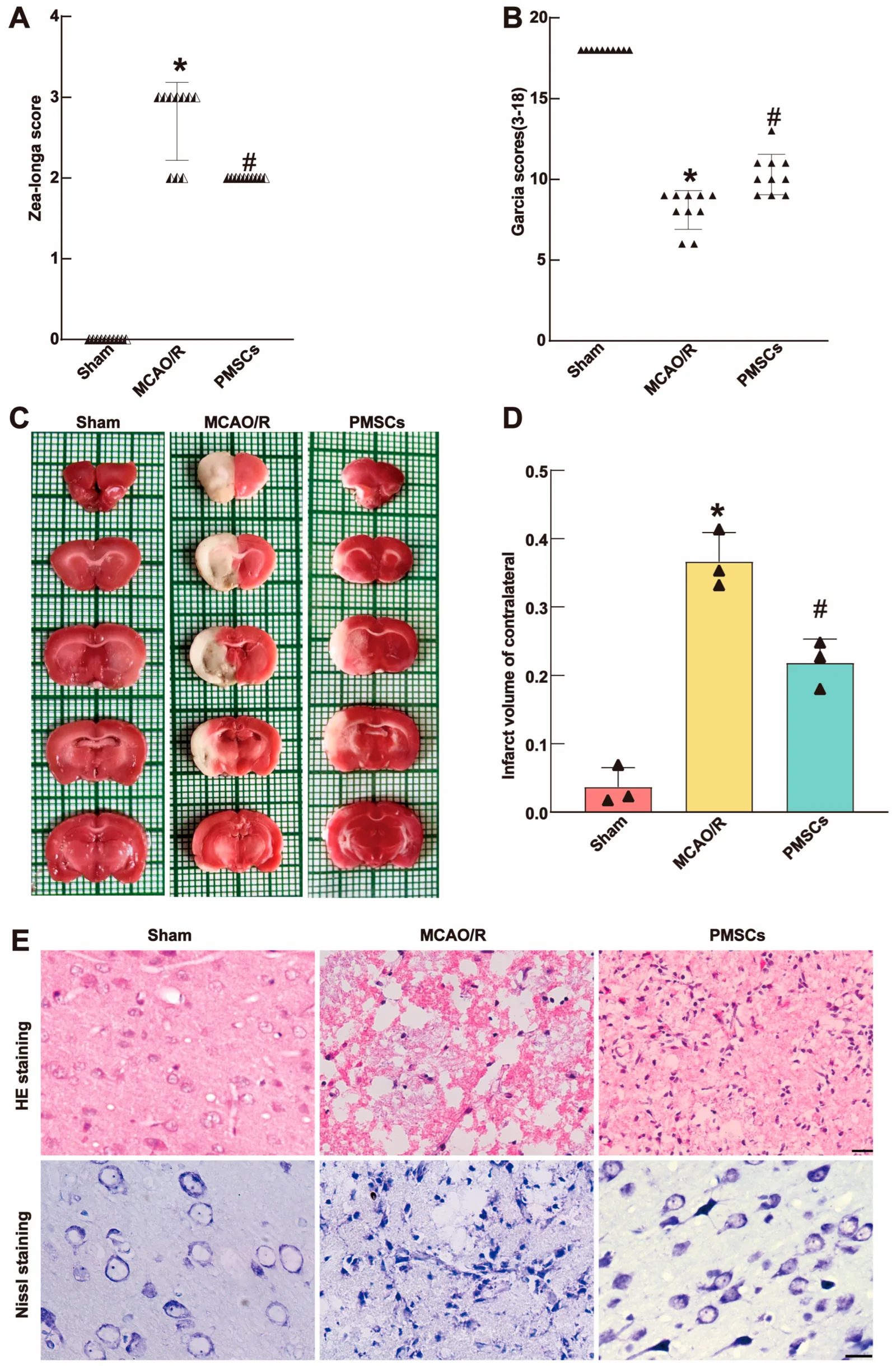
Effects of PMSC transplantation on neurological dysfunction and histopathological damage in MCAO/R rats. (**A**,**B**) Quantitative analysis of the Zea Longa and modified Garcia scoring systems. Behavioral testing was conducted 72 h after surgical procedures in all the cohort (*n* = 10). (**C**) Representative TTC-stained brain slices were captured on day 3 post cell grafting. (**D**) Quantitative measurements of cerebral infarct volume at the same time point (*n* = 3). (**E**) Representative micrographs of HE and Nissl staining were collected post transplantation, with a scale bar of 20 μm (*n* = 5). * *p* < 0.05 relative to the Sham group and ^#^ *p* < 0.05 relative to the MCAO/R group.

To clarify the therapeutic efficacy of PMSC transplantation for cerebral ischemia–reperfusion injury, the Zea Longa scoring system and modified Garcia neurological assessment scale were utilized to evaluate motor function in rats from each experimental group at 72 h post-surgery. Comprehensive neurological evaluations revealed that Sham-operated rats retained intact neurological function, which was confirmed by their baseline Zea Longa scores. In contrast, rats subjected to MCAO/R exhibited significant neurological deficits. Notably, PMSC transplantation remarkably promoted neurological function recovery in MCAO/R rats (*n* = 10, Figure 1A). These therapeutic effects were further corroborated by the results of the modified Garcia scoring system (*n* = 10, Figure 1B).

Three days after MCAO/R model establishment, TTC staining assays revealed uniform red staining throughout the brain tissue sections of rats in the Sham group, with no detectable infarct foci. This finding indicated that the brain tissue had not sustained ischemic injury and maintained normal blood perfusion. In contrast, a distinct pale region was observed in the left hemisphere of the brain tissue from the MCAO/R group, confirming the occurrence of infarction in this area and validating the successful induction of the MCAO/R model. Although a small pale region was also present in the left hemisphere of the PMSC-treated group, its area was notably smaller than that in the MCAO/R group. Quantitative analysis demonstrated that the cerebral infarct volume in the PMSC group was significantly reduced compared with that of the MCAO/R group (*n* = 3, Figure 1C,D).

HE and Nissl staining were employed to evaluate the extent of brain tissue damage in the ischemic region in rats from each experimental group on the third day after PMSC transplantation. HE staining revealed that brain tissues in the Sham group exhibited a well-organized cellular arrangement without obvious structural impairment. In contrast, the MCAO/R group presented sieve-like morphological alterations in the damaged brain tissues, characterized by disorganized cellular alignment, a significant increase in eosinophilic cells and extensive nuclear pyknosis, karyorrhexis, or karyolysis. Compared with the MCAO/R group, the pathological lesions in brain tissues of the PMSC group were significantly mitigated: the damaged region showed a relatively regular cellular arrangement, nuclear morphology approximating that of normal cells, a prominent reduction in pyknosis and karyorrhexis and a scarce presence of eosinophilic cells. Similarly, Nissl staining results demonstrated that in the Sham group, neuronal cells in the brain tissue were arranged in an orderly manner with intact morphology, abundant Nissl bodies and distinct surrounding structures. In the MCAO/R group, the tissue structure in the damaged area was disrupted, displaying a sieve-like pattern accompanied by substantial neuronal loss. The remaining neurons exhibited nuclear pyknosis, karyorrhexis, or karyolysis, along with diminished or absent Nissl bodies. In contrast, neuronal cells in the PMSC group showed morphology comparable to that of normal cells, with a notable reduction in nuclear pyknosis and karyorrhexis and an increased number of Nissl bodies within the cytoplasm (Figure 1E).

### 3.2. Transplantation of PMSCs Alleviated Neuroinflammation and Enhanced Neurotrophic Factor Levels After Cerebral Ischemia–Reperfusion Injury

**Figure 2:**
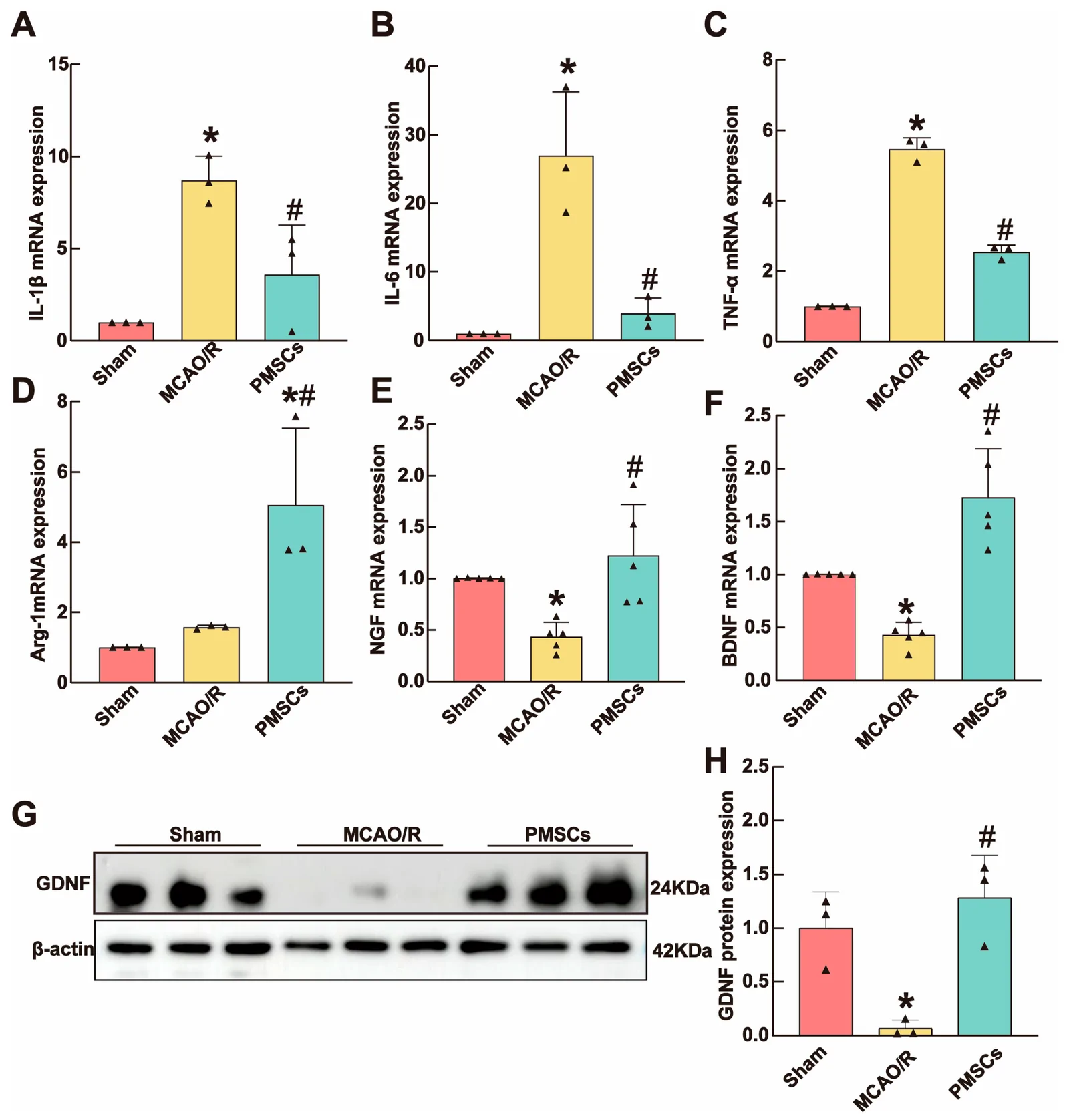
PMSC transplantation regulates cerebral neuroinflammation and neurotrophic factor expression in MCAO/R rats. (**A**–**F**) Relative mRNA levels of IL-1β, IL-6, TNF-α, Arg-1, NGF and BDNF normalized to GAPDH (*n* = 3). (**G**) Representative Western blot bands showing GDNF protein abundance in each group. (**H**) Densitometric quantification of GDNF expression normalized to β-actin (*n* = 3). * *p* < 0.05 vs. Sham group; ^#^ *p* < 0.05 vs. MCAO/R group. Original images of Western blotting can be found in Supplementary Materials.

IL-1β, IL-6, TNF-α and Arg-1 are important inflammation-related factors. We used qRT-PCR to measure the mRNA expression levels of these inflammatory factors in the injured cerebral hemisphere from each group. The results showed that, compared with the Sham group, the mRNA expression of IL-1β, IL-6 and TNF-αwas significantly increased in the MCAO/R group (*n* = 3, Figure 2A–C), indicating that MCAO/R induced an inflammatory response in brain tissue. In comparison with the MCAO/R group, the PMSC group exhibited significantly reduced mRNA expression of IL-1β, IL-6 and TNF-α (*n* = 3, Figure 2A–C), while the expression of the anti-inflammatory factor Arg-1 was markedly upregulated (*n* = 3, Figure 2D). These findings suggest that PMSC transplantation can suppress the inflammatory response following cerebral injury.

The expression levels of neurotrophic factors, including nerve growth factor (NGF), brain-derived neurotrophic factor (BDNF) and glial cell line-derived neurotrophic factor (GDNF), were quantified in each experimental group using qRT-PCR or Western blot analysis. Our results revealed that, compared with the Sham group, the expression levels of NGF (*n* = 3, Figure 2E), BDNF (*n* = 3, Figure 2F) and GDNF (*n* = 3, Figure 2G,H) were significantly downregulated in the MCAO/R group, indicating that ischemia–reperfusion injury causes profound impairment of the neuronal survival microenvironment. In contrast, the expression of NGF (*n* = 3, Figure 2E), BDNF (*n* = 3, Figure 2F) and GDNF (*n* = 3, Figure 2G,H) in the PMSC group was remarkably upregulated, which demonstrates that PMSC transplantation can effectively enhance the production of neurotrophic factors in brain tissues. These findings further confirm the neuroprotective potential of PMSCs in cerebral ischemia–reperfusion injury, providing additional evidence for the therapeutic application of PMSCs in this pathological context.

### 3.3. Transplantation of PMSCs Promotes the Polarization of Astrocytes Towards the A2 Phenotype

GFAP is a marker for astrocytes, inducible nitric oxide synthase (iNOS) is a marker for A1-type astrocytes, and arginase-1 (Arg-1) and S100 calcium-binding protein A10 (S100A10) are markers for A2-type astrocytes. To assess astrocyte polarization, double immunofluorescence staining for GFAP/iNOS and GFAP/Arg-1 was performed on brain tissue sections from each group.

**Figure 3:**
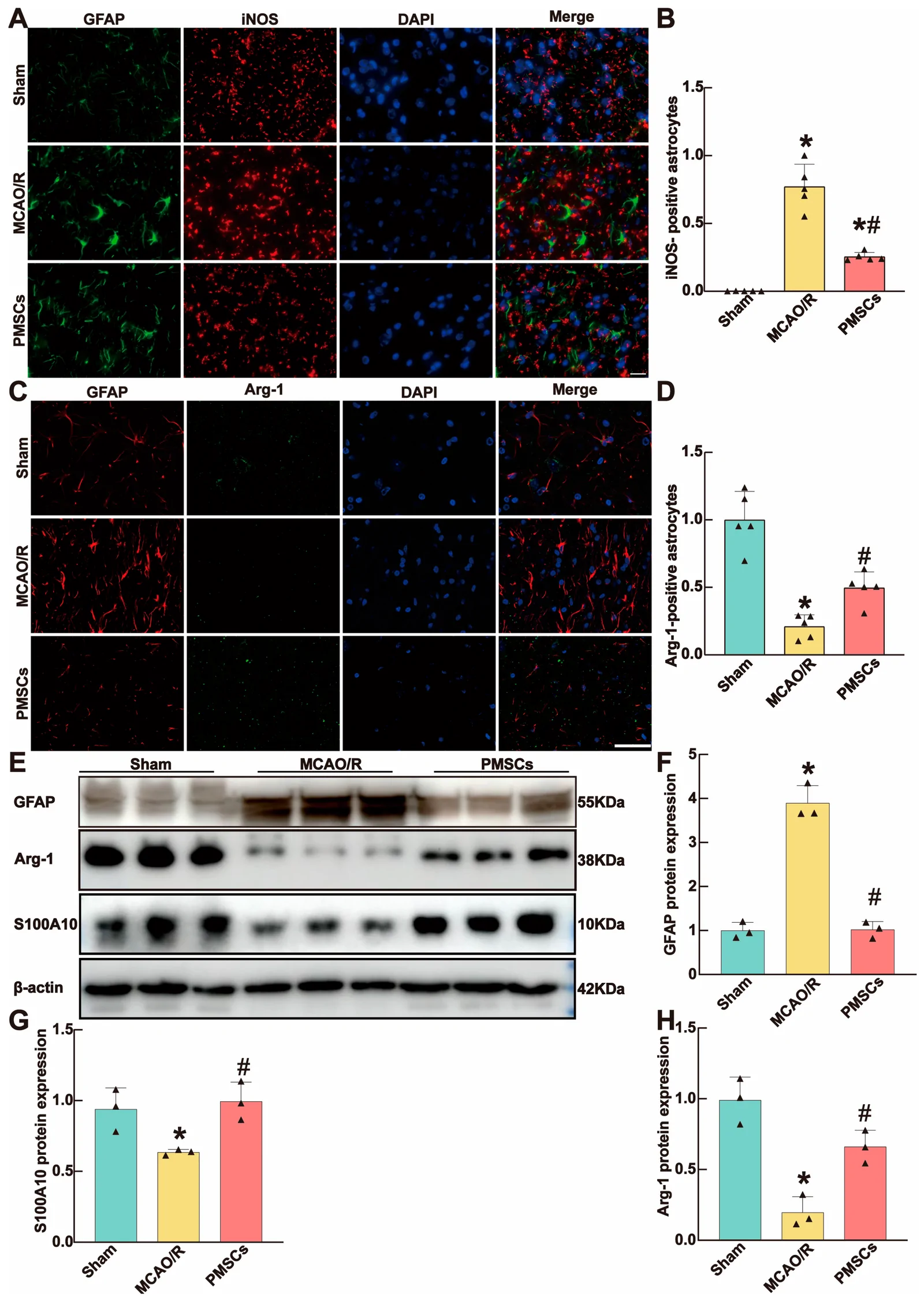
PMSC transplantation modulates astrocytic polarization in the brain of MCAO/R rats. (**A**) Representative immunofluorescence double-staining micrographs of iNOS and GFAP in different experimental groups. Scale bar = 20 μm. (**B**) Statistical quantification of iNOS/GFAP double-positive cell proportions across groups (*n* = 5). (**C**) Representative immunofluorescence images showing Arg-1 and GFAP dual staining in each cohort. Scale bar = 20 μm. (**D**) Quantitative analysis of Arg-1/GFAP double-positive cell numbers for all treatment groups (*n* = 5). (**E**) Exemplary Western blot profiles of GFAP, Arg-1, and S100A10 protein expression in each group. (**F**–**H**) Densitometric quantification of relative GFAP, S100A10 and Arg-1 protein levels, with β-actin serving as the internal normalization reference (*n* = 3). * *p* < 0.05 vs. the Sham group; ^#^ *p* < 0.05 vs. the MCAO/R group. Original images of Western blotting can be found in Supplementary Materials.

As shown in Figure 3A–D, compared with the Sham-operated group, GFAP-positive cells in the MCAO/R group displayed an enlarged soma and increased processes; concurrently, the number of GFAP/iNOS double-positive cells was significantly elevated (*n* = 5, Figure 3A,B), while the number of GFAP/Arg-1 double-positive cells was markedly reduced (*n* = 5, Figure 3C,D). Notably, in the PMSC group, the positive rate of GFAP/iNOS was significantly lower than that in the MCAO/R group (*n* = 5, Figure 3A,B), whereas the positive rate of GFAP/Arg-1 was significantly higher (*n* = 5, Figure 3C,D).

To further validate these observations, Western blot analysis was conducted to quantify the protein expression levels of GFAP, Arg-1 and S100A10 in rat brain tissue from each group. Consistent with the immunofluorescence results, the expression levels of Arg-1 (*n* = 3, Figure 3E,H) and S100A10 (*n* = 3, Figure 3E,G) in the MCAO/R group were significantly downregulated compared with the Sham-operated group, and PMSC transplantation significantly upregulated the expression of these proteins relative to the MCAO/R group (*n* = 3, Figure 3E–H). Whereas the expression levels of GFAP in the MCAO/R group were significantly upregulated compared with the Sham-operated group, PMSC transplantation significantly downregulated the expression level relative to the MCAO/R group (*n* = 3, Figure 3F).

Taken together, these findings indicate that PMSC transplantation may exert neuroprotective effects against MCAO/R-induced neural injury by promoting the A2 polarization of astrocytes.

### 3.4. Transplantation of PMSCs Attenuated the Expression of TGF-β1 Signaling Pathway-Related Factors in the Brains of MCAO/R Rats

qRT-PCR and Western blot analyses were employed to determine the mRNA and protein expression levels of TGF-β signaling pathway-related markers in the cerebral ischemic penumbra of rats across all experimental groups.

**Figure 4:**
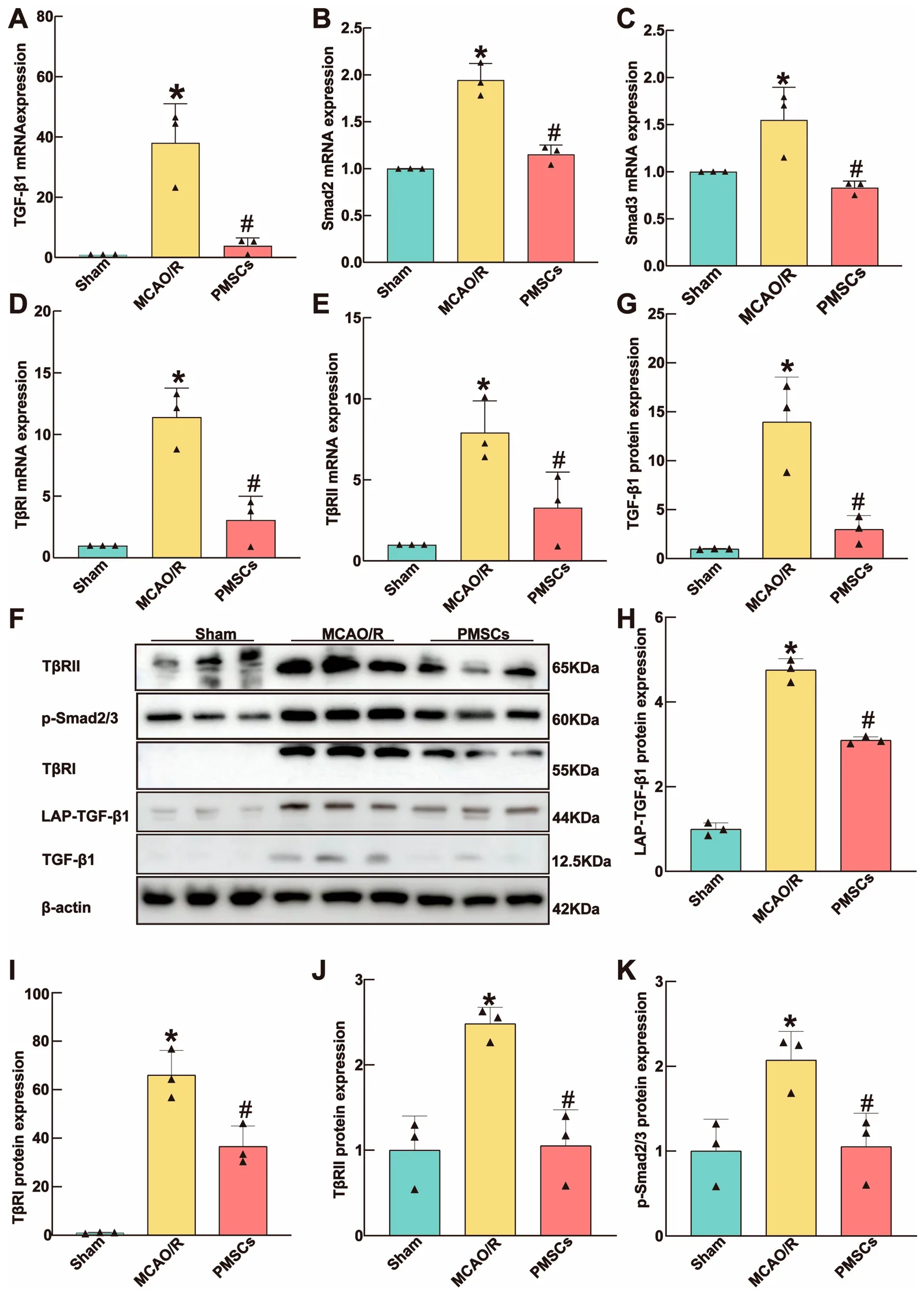
PMSC transplantation regulates the expression of key molecules associated with the cerebral TGF-β1 signaling pathway in MCAO/R rats. (**A**–**E**) Relative transcript levels of TGF-β1, TβRI, TβRII, Smad2, and Smad3 in distinct groups, with all mRNA values normalized to GAPDH (*n* = 3). (**F**) Representative Western blot bands showing the expression profiles of TGF-β1, LAP-TGF-β1, TβRI, TβRII, and p-Smad2/3 across experimental cohorts. (**G**–**K**) Densitometric quantification of TGF-β1, LAP-TGF-β1, TβR I, TβR II, and p-Smad2/3 protein abundances. All protein data were calibrated using β-actin as the internal reference (*n* = 3). * *p* < 0.05 vs. the Sham group; ^#^ *p* < 0.05 vs. the MCAO/R group. Original images of Western blotting can be found in Supplementary Materials.

The mRNA expression levels of TGF-β1, TβRI, TβRII, Smad2 and Smad3 were maintained at low basal levels in the Sham group. In comparison to the Sham group, MCAO/R surgery led to a significant upregulation of TGF-β1 (*n* = 3, Figure 4A), TβRI (*n* = 3, Figure 4D), TβRII (*n* = 3, Figure 4E), Smad2 (*n* = 3, Figure 4B) and Smad3 (*n* = 3, Figure 4C) mRNA levels. Notably, PMSC transplantation markedly reversed these elevations: the mRNA expression of the aforementioned genes in the PMSC group was significantly lower than that in the MCAO/R group (*n* = 3, Figure 4A–E).

Consistent with the qRT-PCR findings, Western blot results showed that the protein expression levels of TGF-β1, LAP-TGF-β1, TβRI, TβRII and p-Smad2/3 were minimal in the Sham group. Relative to the Sham group, MCAO/R induced a significant increase in the protein levels of these factors. In contrast, PMSC transplantation significantly reduced the protein expression of TGF-β1, LAP-TGF-β1, TβRI, TβRII and p-Smad2/3 compared with the MCAO/R group (*n* = 3, Figure 4F–K).

Taken together, these results indicate that cerebral ischemia–reperfusion injury activated the TGF-β1 signaling pathway in the brain, and PMSC transplantation could effectively inhibit the activation of this pathway.

### 3.5. PMSC Treatment Promoted Interleukin-1β-Treated Astrocyte Polarization from the A1 to A2 Subtype

IL-1β is a key pro-inflammatory cytokine that contributes to the pathological progression of cerebral ischemia–reperfusion injury [16]. To mimic the in vitro inflammatory microenvironment of post cerebral ischemia–reperfusion, we established an IL-1β-induced astrocyte activation model. IL-6, TGF-β, TNF-α, iNOS, complement component 3 (C3) and GFAP are well-recognized molecular markers linked to the activation of neurotoxic A1 reactive astrocytes [17,18]. In contrast, Arg-1 and S100A10 serve as characteristic markers of neuroprotective A2 reactive astrocytes [19]. Following 24 h of co-culturing PMSCs with IL-1β-stimulated astrocytes, we detected the expression levels of A1- and A2-specific markers in each group, while comparing the regulatory effects of PMSCs with those of SB-431542, a selective inhibitor of the TGF-β1 signaling pathway.

**Figure 5:**
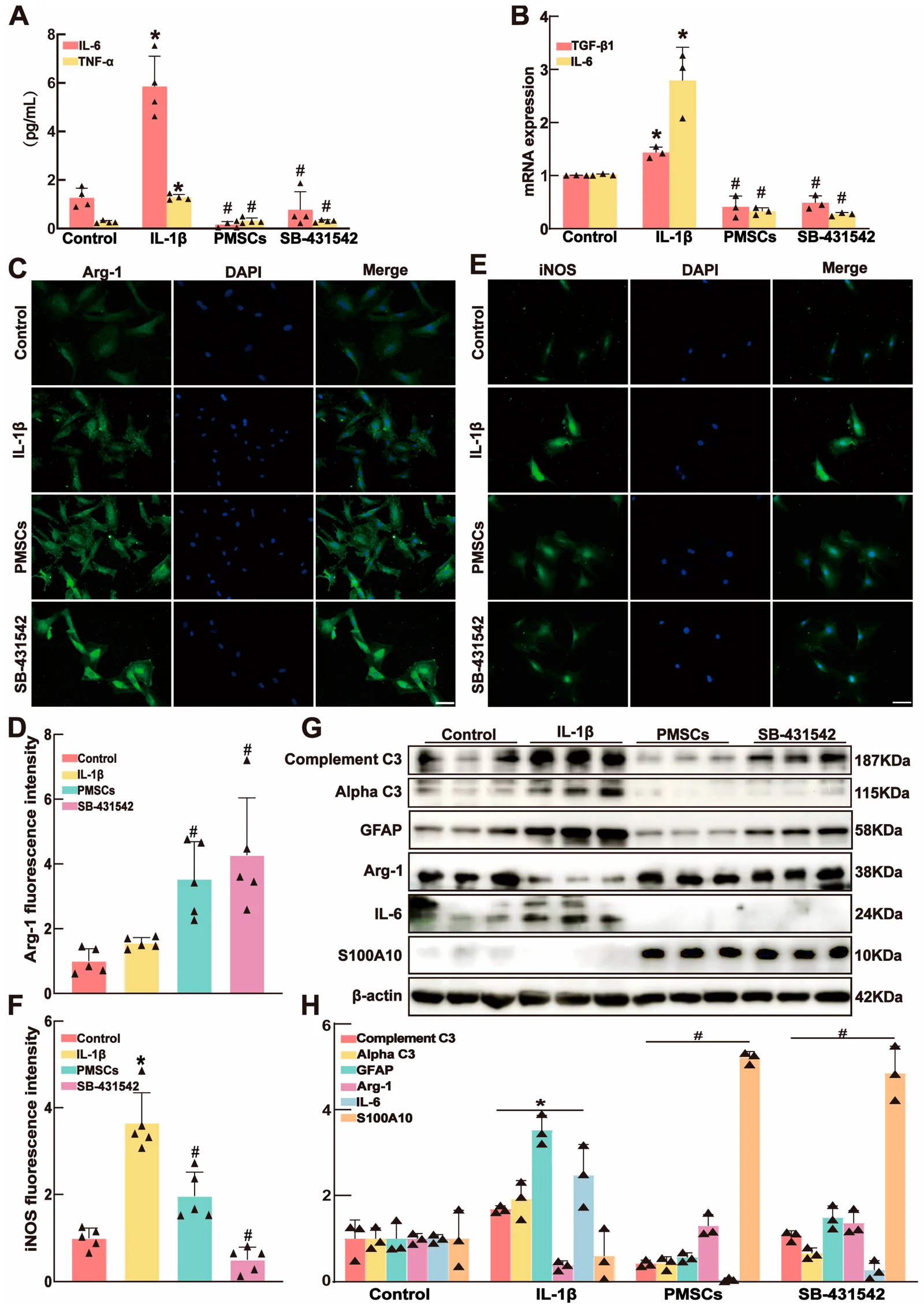
PMSC treatment modulates astrocytic polarization in an IL-1β-stimulated astrocyte activation cell model. (**A**) ELISA quantification of secreted IL-6 and TNF-α in cell culture supernatants (*n* = 4). (**B**) Relative transcript abundances of IL-6 and TGF-β1 normalized to GAPDH (*n* = 3). (**C**,**E**) Representative immunofluorescence micrographs of Arg-1 and iNOS signals in astrocytes. Scale bar = 20 μm. (**D**,**F**) Quantified fluorescence intensity of Arg-1- and iNOS-positive astrocytes (*n* = 5). (**G**) Representative Western blot bands showing protein profiles of C3, αC3, GFAP, IL-6, Arg-1 and S100A10. (**H**) Densitometric quantification of target proteins normalized to β-actin (*n* = 3). * *p* < 0.05 vs. normal control group; ^#^ *p* < 0.05 vs. IL-1β group. Original images of Western blotting can be found in Supplementary Materials.

ELISA was performed to quantify IL-6 and TNF-α secretion in cell culture supernatants. The results showed that IL-1β stimulation significantly increased IL-6 and TNF-αlevels compared with the normal control group, whereas PMSC co-culture led to a marked reduction in the secretion of these two pro-inflammatory cytokines relative to the IL-1β group. SB-431542 exerted a suppressive effect on IL-6 and TNF-α production comparable to that of PMSCs (*n* = 4, Figure 5A).

Compared with the normal control group, the mRNA expression levels of IL-6 and TGF-β1 were significantly upregulated in the IL-1β-treated group (*n* = 3, Figure 5B). In contrast, IL-6 and TGF-β1 mRNA levels were remarkably downregulated in the PMSC co-culture group relative to the IL-1β group (*n* = 3, Figure 5B), and the inhibitory effect of SB-431542 treatment was consistent with that of PMSC co-culture (*n* = 3, Figure 5B).

Immunofluorescence staining was employed to assess the expression of Arg-1 and iNOS in astrocytes. The fluorescence intensity of Arg-1-positive cells seemed slightly increased in the IL-1β group compared to the normal control group, but showed no statistical significance, whereas both PMSC co-culture and SB-431542 treatment significantly increased the fluorescence intensity compared with the IL-1β group (*n* = 5, Figure 5C,D). Meanwhile, the fluorescence intensity of iNOS-positive cells was notably reduced in both the PMSC and SB-431542 groups relative to the IL-1β group (*n* = 5, Figure 5E,F).

Western blot analysis was carried out to determine the protein expression levels of complement C3, alpha C3, GFAP, IL-6, Arg-1 and S100A10. Compared with the normal control group, IL-1β stimulation significantly upregulated the protein levels of complement C3, its active fragment alpha C3, GFAP and IL-6, while significantly reducing Arg-1 protein expression. In contrast, both PMSC co-culture and SB-431542 administration significantly downregulated the protein expression of complement C3, alpha C3, GFAP and IL-6 and upregulated the protein levels of Arg-1 and S100A10, compared with the IL-1β group (*n* = 3, Figure 5G,H).

### 3.6. PMSC Treatment Attenuated the Expression of TGF-β Signaling Pathway-Related Factors in Astrocytes

**Figure 6:**
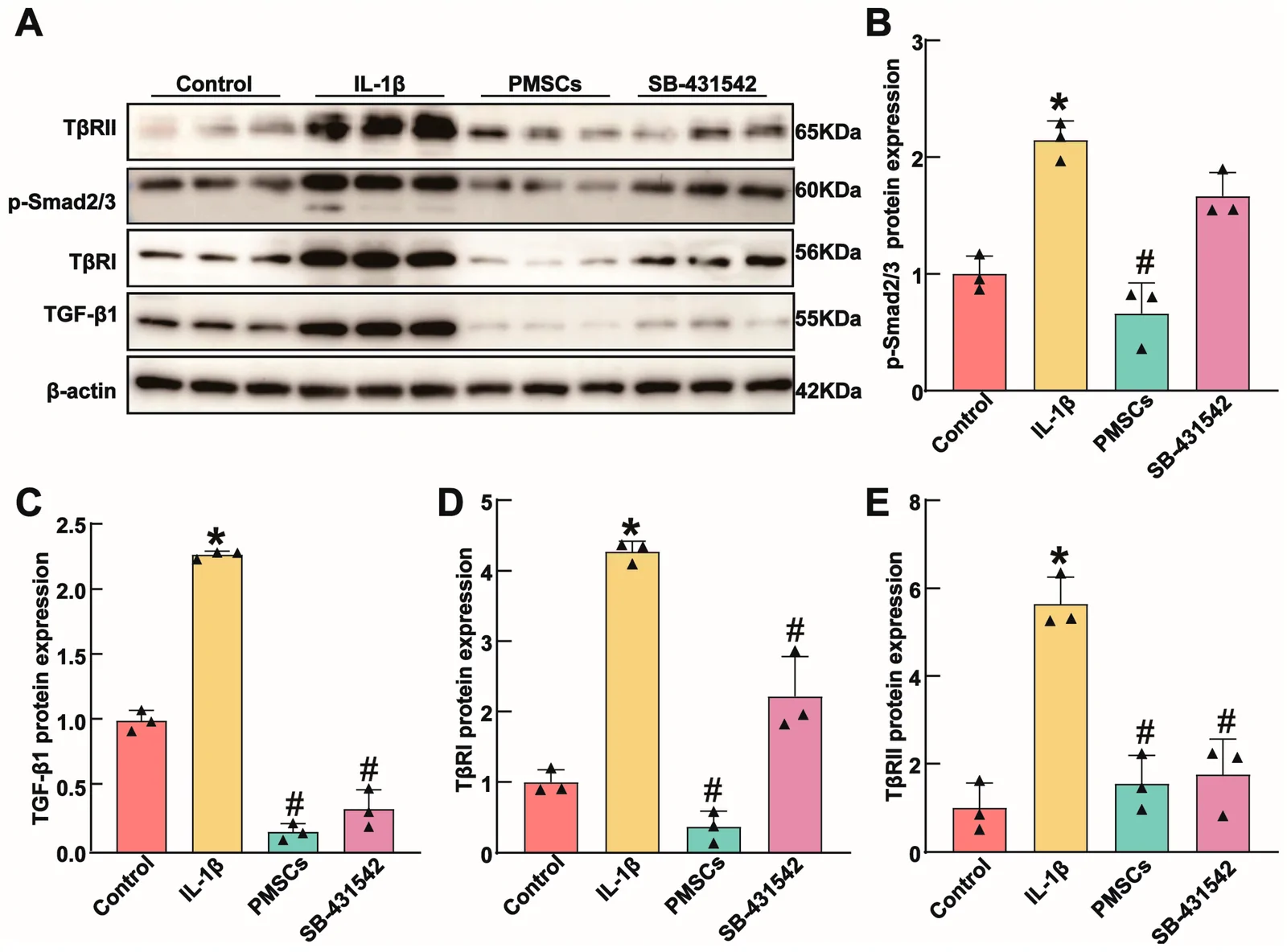
PMSC intervention modulates the expression of core TGF-β1 signaling components in an IL-1β-induced astrocyte activation model. (**A**) Representative Western blot analyses showing the protein expression patterns of TGF-β1, TβRI, TβRII, and p-Smad2/3 in different treatment groups. (**B**–**E**) Quantitative densitometry of TGF-β1, TβRI, TβRII, and p-Smad2/3 protein levels, with β-actin serving as the internal reference for normalization (*n* = 3). * *p* < 0.05 versus the normal control group; ^#^ *p* < 0.05 versus the IL-1β-stimulated group. Original images of Western blotting can be found in Supplementary Materials.

To further explore the regulatory effect of PMSCs on the TGF-β signaling pathway in astrocytes activated by IL-1β, we detected the protein expression levels of TGF-β1, TβRI, TβRII, p-Smad2 and p-Smad3 in cells from each experimental group. The results showed that, compared with the normal control group, the protein expression levels of TGF-β1, TβRI, TβRII, and p-Smad2/3 in the IL-1β-treated group were significantly upregulated (*n* = 3, Figure 6A–E), suggesting that IL-1β treatment effectively activates the TGF-β signaling pathway in astrocytes. In contrast to the IL-1β group, the protein expression levels of TGF-β1, TβRI and TβRII were markedly downregulated in both the PMSC-treated group and the SB-431542-treated group, and the levels of p-Smad2/3 were downregulated only in the PMSC-treated group (*n* = 3, Figure 6A–E).

## 4. Discussion

Stroke is a major contributor to global death and disability, and is predominantly caused by ischemic events [20]. Consequently, discovering and optimizing effective therapies remains an area of intense investigation [21].

The pathophysiology of CIRI involves multiple cell types and biological pathways [22]. During the acute phase of cerebral ischemia, local hypoxia–ischemia, acidosis and metabolic derangements drive extensive neuronal apoptosis and necrosis [23]. Upon reperfusion, the influx of oxygen and nutrients with restored cerebral blood flow leads to a surge in reactive oxygen species [24] and pro-inflammatory cytokines [25]. Several studies have demonstrated that PMSCs not only possess potent immunomodulatory properties [26] but also demonstrate the ability to suppress inflammatory responses [14,27]. PMSCs orchestrate inflammatory resolution and tissue regenerative recovery through an array of divergent signaling regulatory axes, corroborating previous investigative evidence [28,29,30]. In the present study, we observed that PMSC transplantation significantly improved motor function in MCAO/R rats, markedly reduced infarct volume and alleviated neuronal damage. Specifically, in vivo measurements confirmed that PMSC grafting profoundly attenuated the abundance of cerebral pro-inflammatory mediators IL-1β, IL-6 and TNF-α in the brain parenchyma of MCAO/R rats, while simultaneously upregulating the mRNA expression of anti-inflammatory markers such as Arg-1. These changes suggest that PMSCs effectively mitigate brain tissue injury by modulating the inflammatory milieu. Moreover, PMSC transplantation significantly elevated the expression of these neurotrophic factors, indicating that PMSCs promote the release of neurotrophic support molecules that are critical for neuronal survival and functional recovery. These findings provide compelling evidence that PMSCs exert significant neuroprotective effects in cerebral ischemia–reperfusion injury by attenuating post-neuroinflammation, ultimately contributing to the preservation of neuronal function and improvement of neurological outcomes. Notably, PMSCs also exert pleiotropic effects encompassing immunomodulation, angiogenesis and anti-apoptosis; these multifaceted biological actions may jointly participate in post-ischemic functional restoration.

Astrocytes are among the most important glial cell types in the central nervous system and are extensively involved in maintaining neural microenvironment homeostasis, regulating neurotransmission and supporting neural repair [31]. Following cerebral ischemia, astrocytes are robustly activated to become reactive astrocytes, characterized by cellular hypertrophy, elongated processes and the release of inflammatory mediators and neurotrophic factors [32]. During the pathophysiological process of CIRI, the activation and polarization states of astrocytes play pivotal roles in both neuronal injury and repair [33]. Astrocytic polarization comprises two principal phenotypes: A1 and A2. A1 astrocytes exert neurotoxic effects soon after injury via the secretion of pro-inflammatory cytokines, whereas A2 astrocytes promote tissue repair by releasing anti-inflammatory factors and neurotrophic factors [34]. This work validates that PMSC grafting drives an astrocytic shift to anti-inflammatory A2 subtypes in MCAO/R cerebral parenchyma. In vitro, IL-1β-treated astrocytes exhibited marked GFAP upregulation, which occurs after astrocytic activation and plays an important role in neuroinflammatory processes [35], and significantly increased expression of IL-6, TGF-β, TNF-α, iNOS and C3. All of these results indicated an A1-dominant state. Meanwhile, PMSC treatment effectively suppressed A1-type activation and stimulated a shift toward the A2 phenotype. Collectively, these findings demonstrate that PMSCs mitigate neuroinflammation in the ischemic brain by inhibiting A1 astrocyte activation and promoting A2 polarization.

Astrocyte activation is orchestrated by a complex network of signaling pathways [36,37], among which the TGF-β pathway plays a pivotal role [38]. The TGF-β/Smad cascade is a central regulator of multiple critical cellular processes [39], including proliferation, activation and inflammatory responses [40]. TGF-β1, along with its receptors TβRI and TβRII and the downstream signaling molecules of the Smad family, exerts pivotal regulatory effects on a wide spectrum of immune responses and cell migration processes. Upon binding to its cognate receptors TβRI and TβRII, TGF-β1 triggers the activation of downstream Smad2 and Smad3, which in turn modulates cell proliferation, differentiation and anti-inflammatory responses. Phosphorylated Smad2 (p-Smad2) and phosphorylated Smad3 (p-Smad3) are the activated phosphorylated forms of Smad2 and Smad3, respectively, and their expression levels are commonly used as hallmark indicators for the activation status of the TGF-β1 signaling pathway [38]. The TGF-β/Smad pathway has been implicated in astrocyte activation [10] and GFAP expression [38]. In the present study, we observed a marked upregulation of TGF-β1 and activation of the TGF-β1 pathway in brain tissue following CIRI. PMSC intervention suppressed the expression of TGF-β1/Smad-related factors, suggesting that PMSCs may mitigate neuroinflammation in CIRI by reprogramming TGF-β1/Smad signaling. In vitro, activated A1 astrocytes exhibited robust TGF-β1/Smad pathway activation. Treatment with PMSCs significantly reduced the levels of key pathway components, including TβRI, TβRII, p-Smad2 and p-Smad3. In contrast, treatment with SB431542 only induced a moderate downward trend in p-Smad2/3 abundance, yet this suppressive change failed to reach statistical significance relative to the IL-1β-treated group. This contrast in statistical outcomes may hint at possible mechanistic distinctions between PMSC paracrine effects and direct ALK5 pharmacological inhibition. Collectively, these data imply that A1 astrocyte activation is closely associated with TGF-β1/Smad signaling and that PMSCs suppress A1 activation, thereby attenuating neuroinflammation in CIRI by inhibiting this pathway.

There are some limitations in this study, and additional experiments will be required in subsequent research. First, only limited A1/A2 markers were used to phenotype post-ischemic astrocytes in this study. Astrogliosis constitutes a continuous spectrum, demanding unbiased subtyping; multidimensional data are necessary to establish the distinctiveness of astrocyte phenotypes in our future study. Second, the current results regarding mechanistic evidence remain largely associative and need to be verified in a series of follow-up experiments. Third, we used a sole experimental model without setting long-term observation endpoints, and no behavioral testing indicators were applied for cognitive or emotional function to fully evaluate post-stroke recovery. Finally, despite the promising neuroprotective capacity of PMSCs observed in preclinical ischemic models in this study, poor survival, allogeneic immune risks, inconsistent preparation standards and long-term safety issues hinder their application in clinical brain injury.

## 5. Conclusions

Our results suggest that PMSC transplantation may mitigate MCAO/R-triggered neuroinflammation via TGF-β1 pathway regulation, potentially shifting reactive A1 astrocytes to the protective A2 phenotype.

## Data Availability

The original contributions presented in this study are included in the article/Supplementary Material. Further inquiries can be directed to the corresponding author(s).

## Supplementary Materials

The following supporting information can be downloaded at: https://www.mdpi.com/article/10.3390/biom16081103/s1, File S1: Original images of Western blotting.

## Abbreviations

The following abbreviations are used in this manuscript:

Arg-1	Arginase-1
BDNF	Brain-derived neurotrophic factor
CIRI	Cerebral ischemia–reperfusion injury
GDNF	Glial cell line-derived neurotrophic factor
GFAP	Glial fibrillary acidic protein
HE	Hematoxylin and eosin
iNOS	Inducible nitric oxide synthase
MCAO/R	Middle cerebral artery occlusion and reperfusion
MSC	Mesenchymal stem cell
NGF	Nerve growth factor
PMSCs	Placental mesenchymal stem cells
TGF-β	Transforming growth factor-β
TTC	2,3,5-triphenyltetrazolium chloride
