# Supplementary material for: Placental Mesenchymal Stem Cells Promote the Polarization of Astrocytes Towards the A2 Phenotype by Modulating the TGF-β1 Signaling Pathway in Cerebral Ischemia–Reperfusion Rats
Source: Biomolecules. 2026 Jul 28;16(8):1103. doi: 10.3390/biom16081103 (PMC13510269; doi:10.3390/biom16081103)
Supplement: Supplementary file 1 [file biomolecules-16-01103-s001.zip › biomolecules-4399197-supplementary.pdf]

File S1. Original images of Western blotting

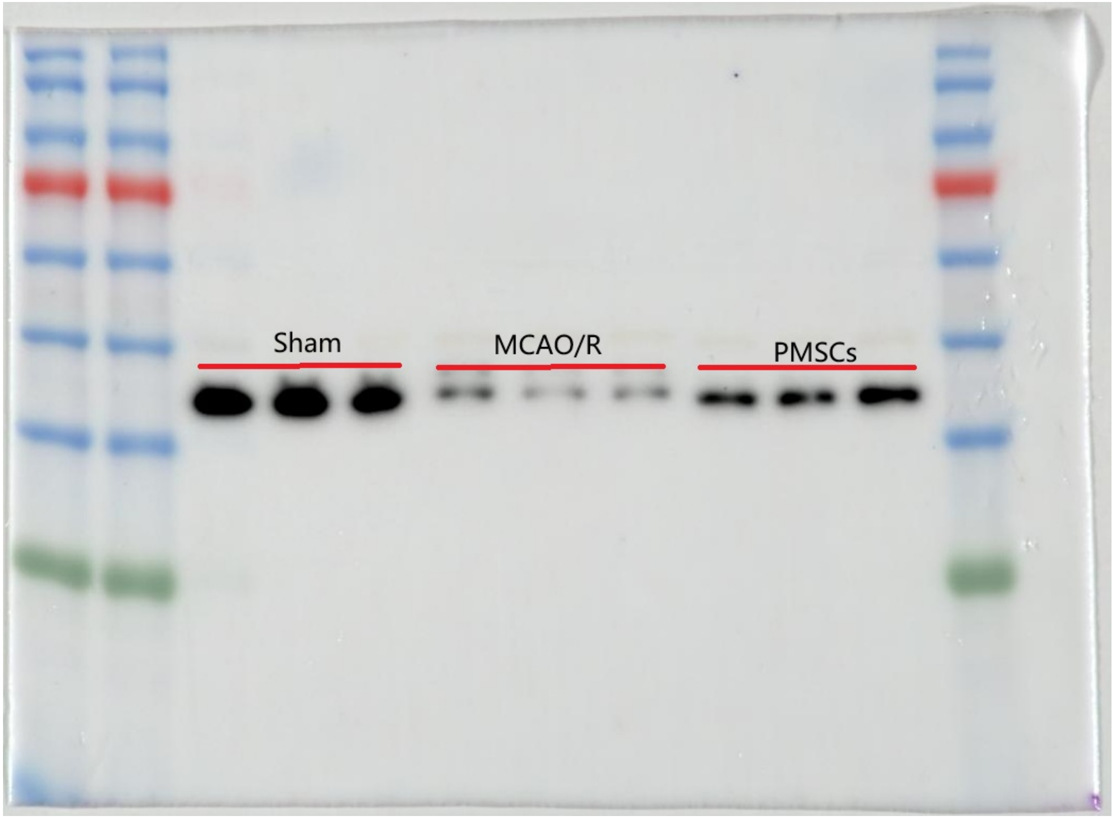

uncropped blot image of Arg-1 in Fig. 3E

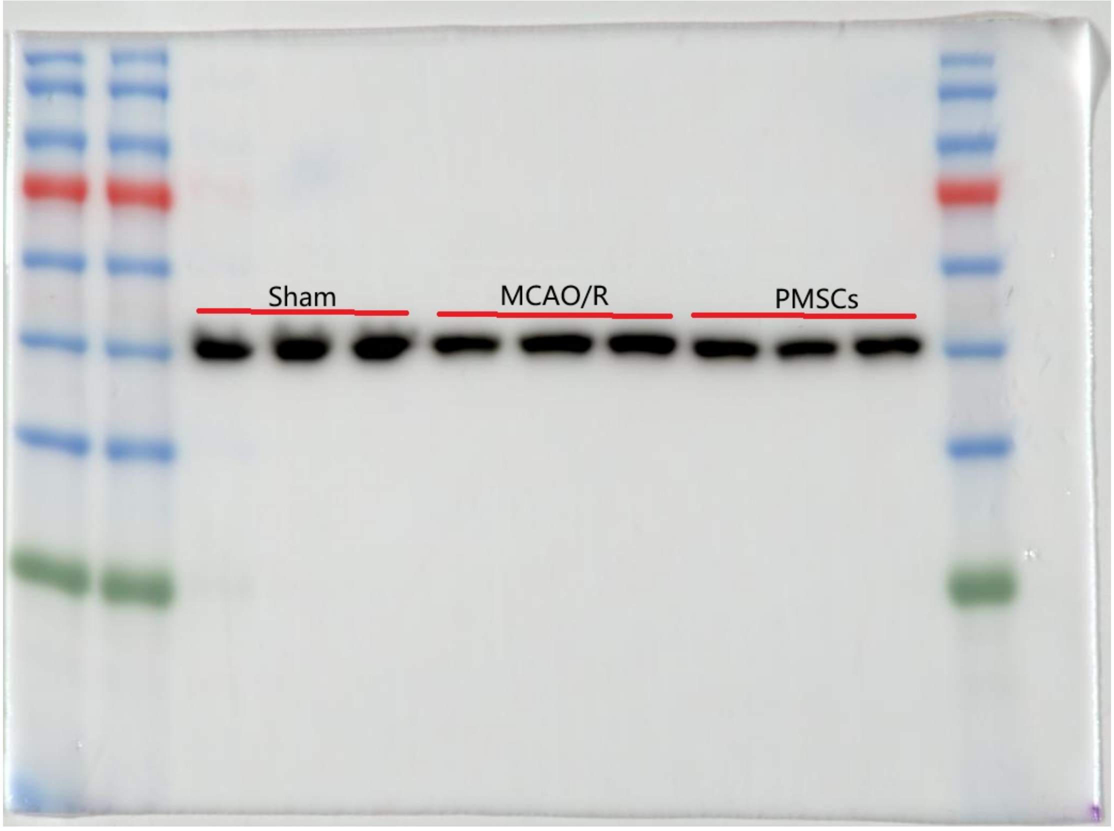

uncropped blot image of β-actin in Fig. 3E-relative to Arg-1

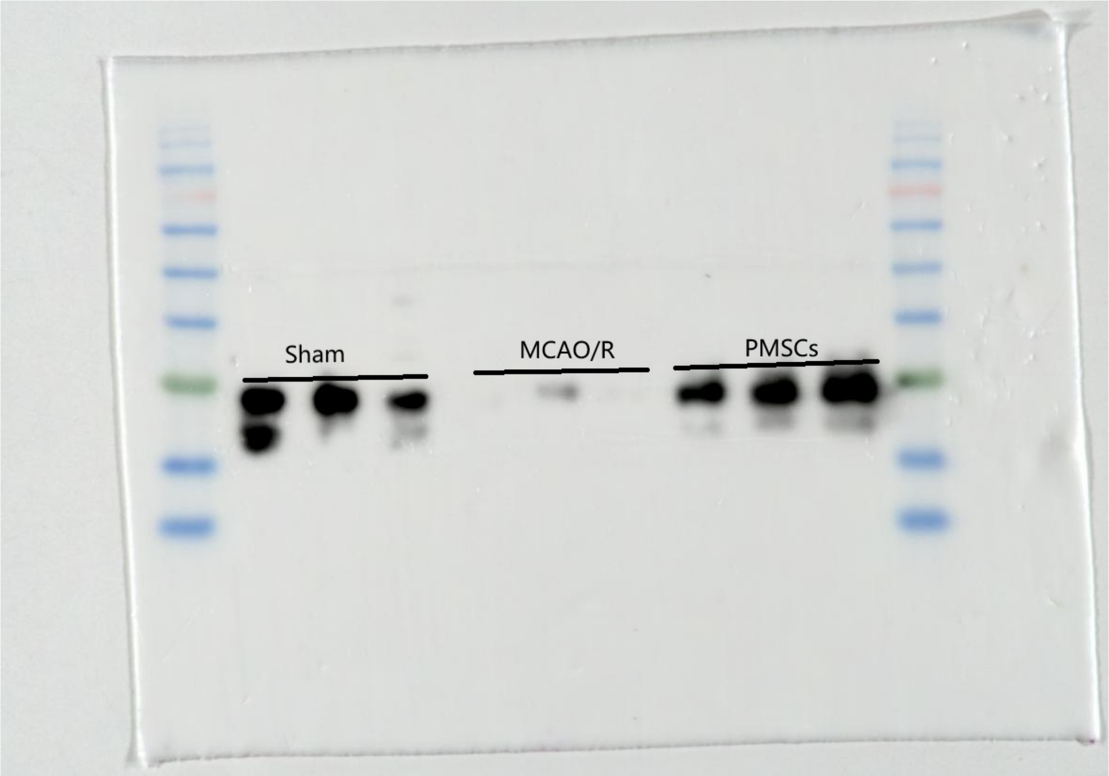

uncropped blot image of GDNF in Fig. 2G

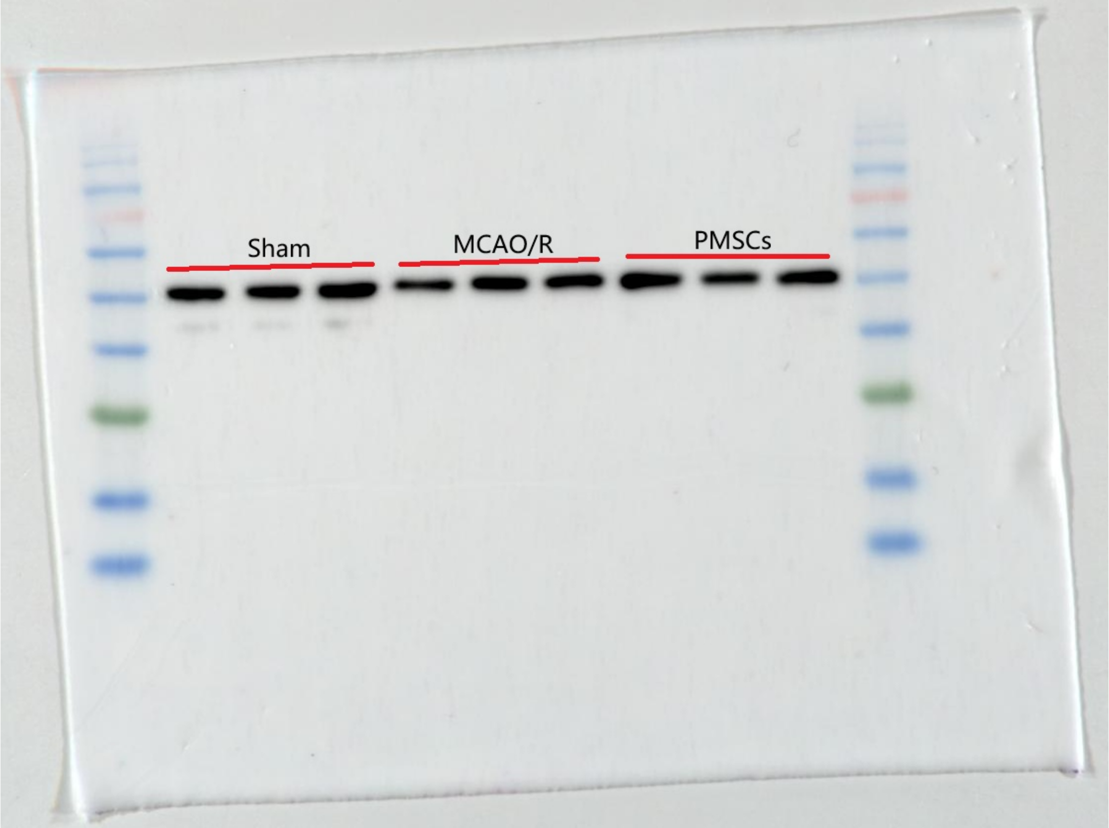

uncropped blot image of β-actin in Fig. 2G

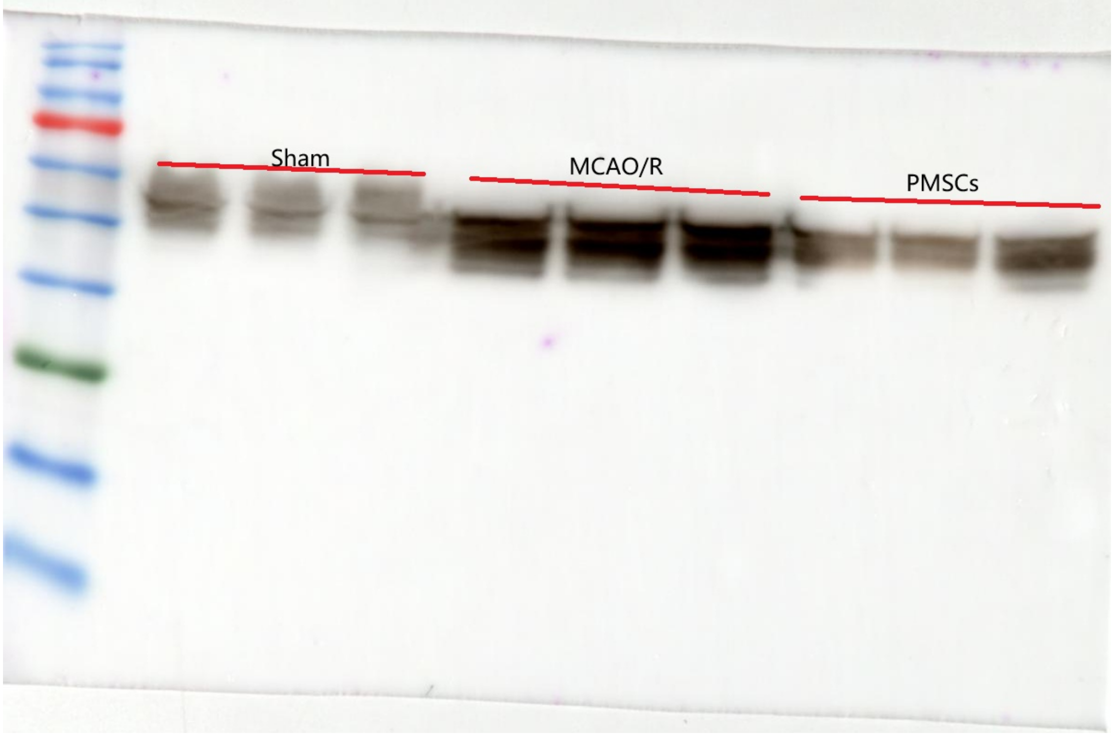

uncropped blot image of GFAP in Fig. 3E

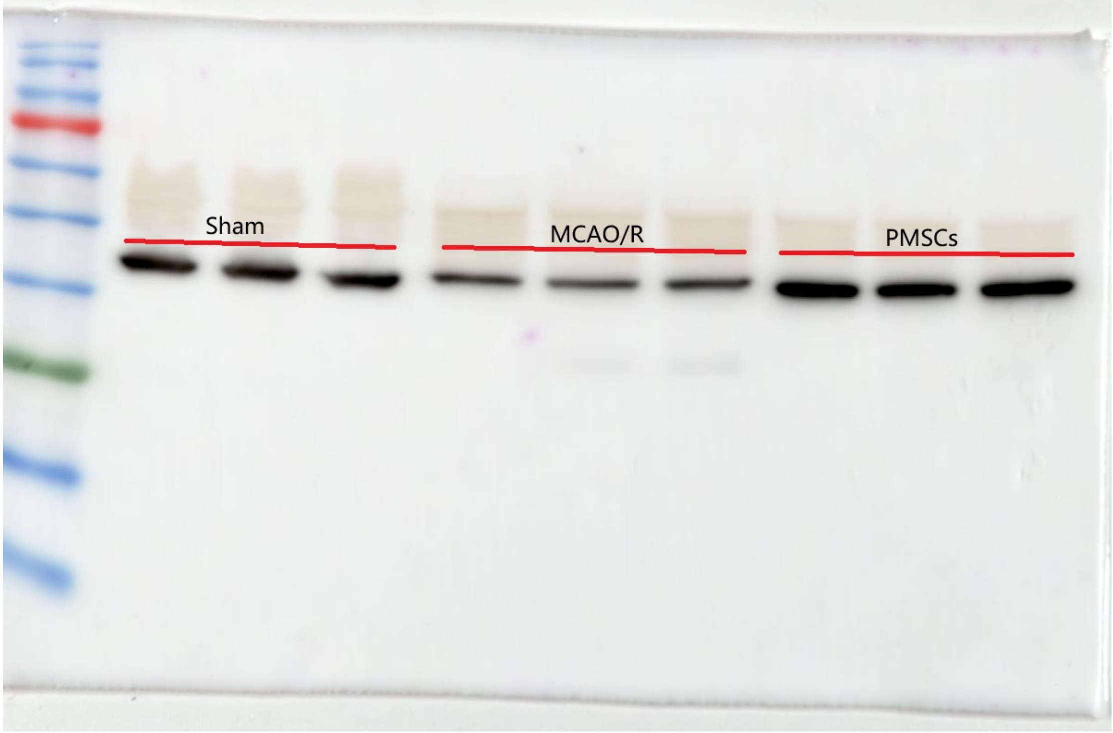

uncropped blot image of β-actin in Fig. 3E-relative to GFAP

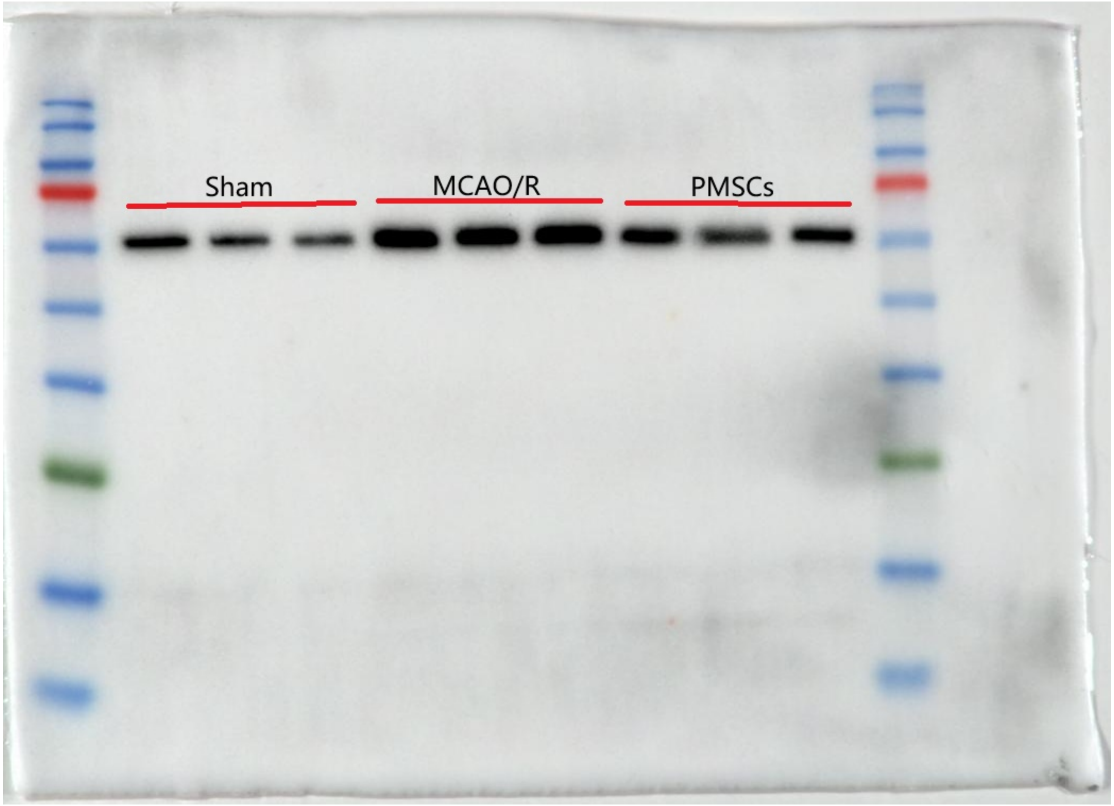

uncropped blot image of p-Smad2.3 in Fig. 4F

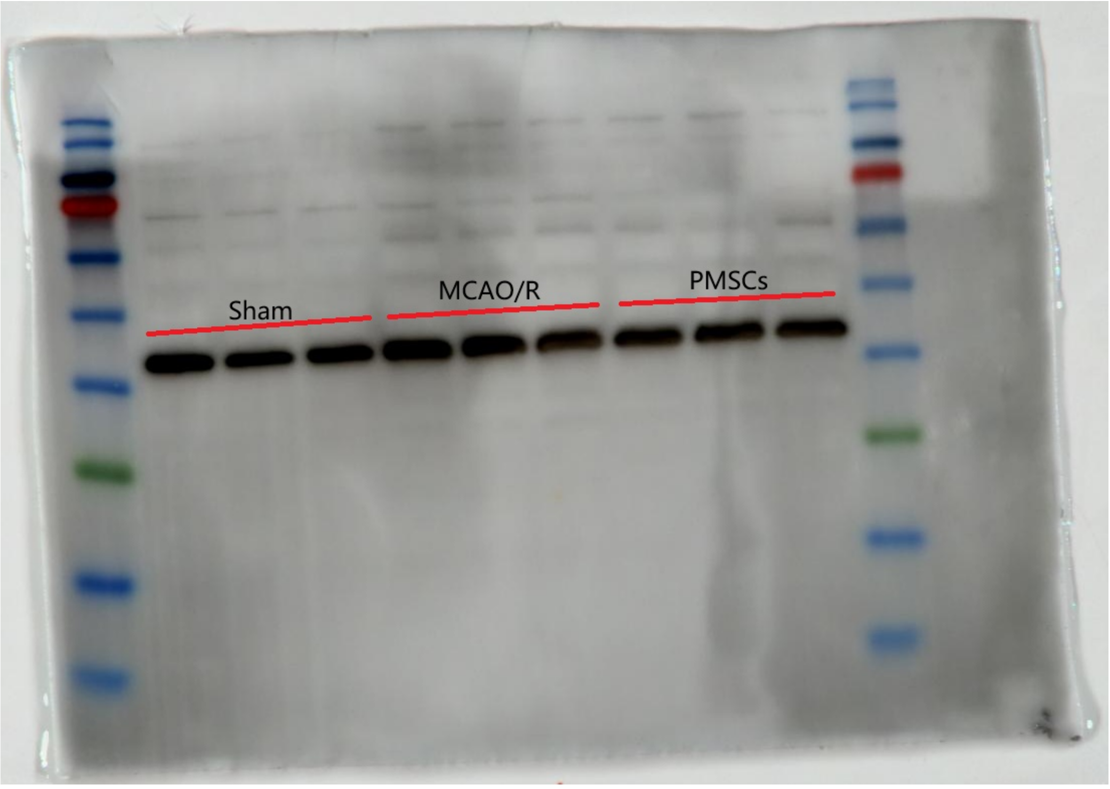

uncropped blot image of GAPDH in Fig. 4F relative to p-smad2.3

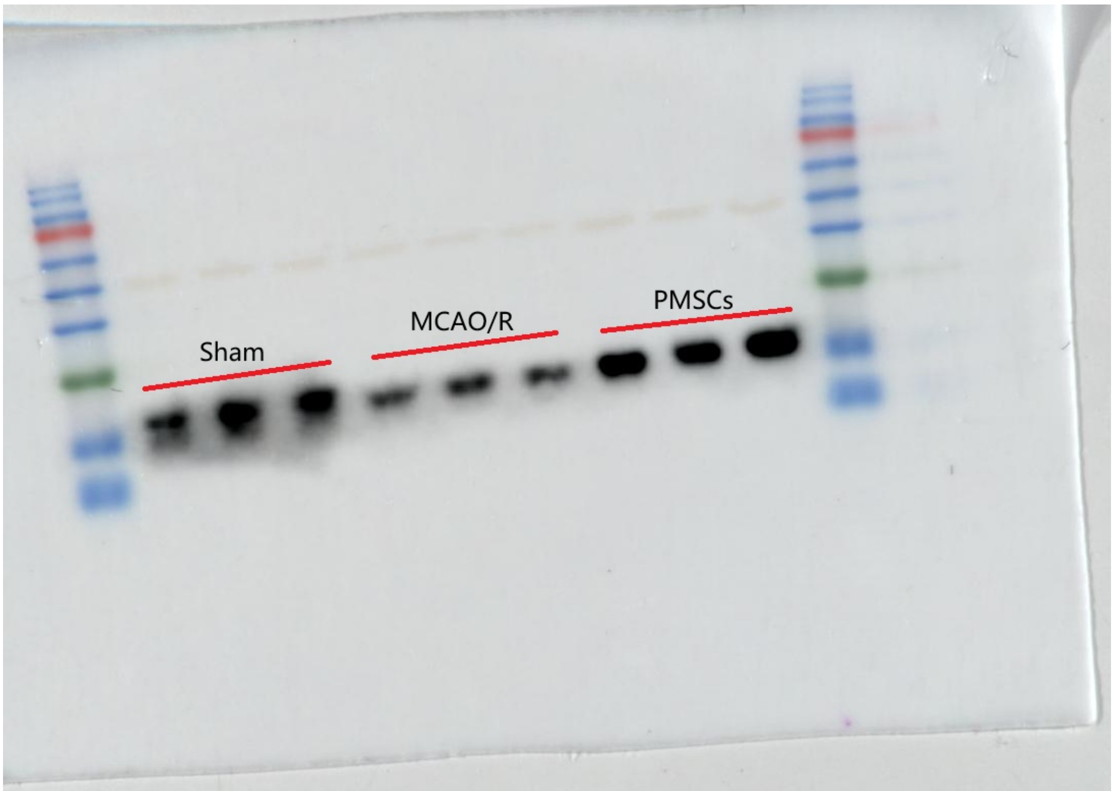

uncropped blot image of S100A10 in Fig. 3E-labeled

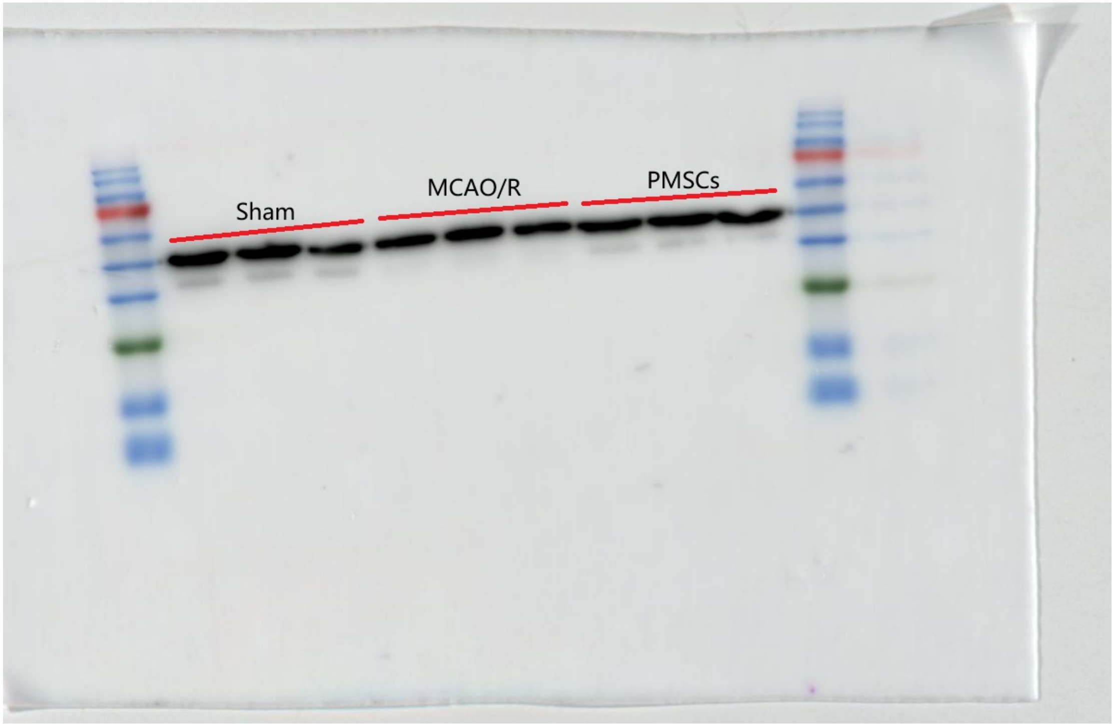

uncropped blot image of β-actin in Fig. 3E-relative to s100a10

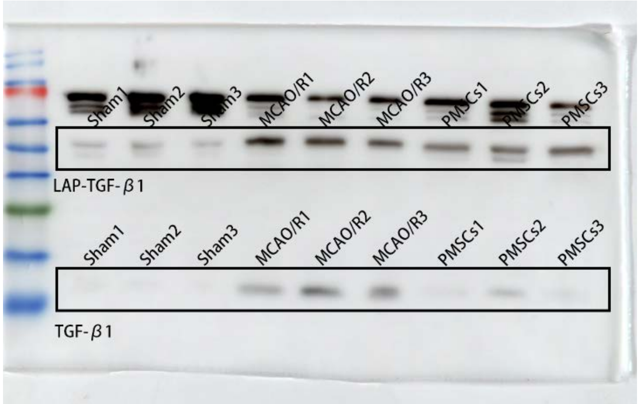

uncropped blot image of TGF-β1 and LAP-TGF-β1 in Fig.4F

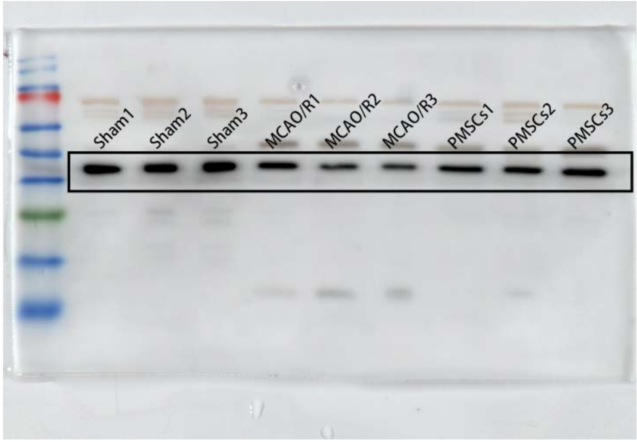

uncropped blot image of GAPDH in Fig.4F-relative to TGFB1 and LAPITGFB1

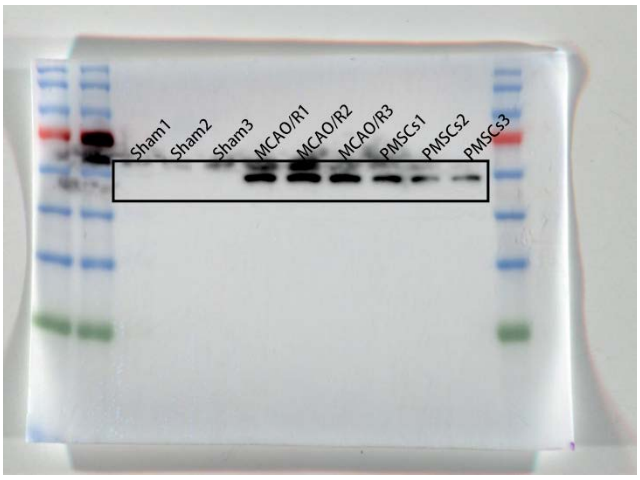

uncropped blot image of TβR1in Fig.4F

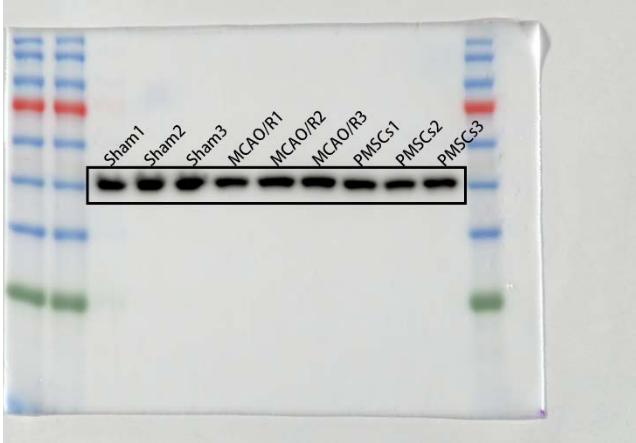

uncropped blot image of beta-actin in Fig.4F-relative to TBR1

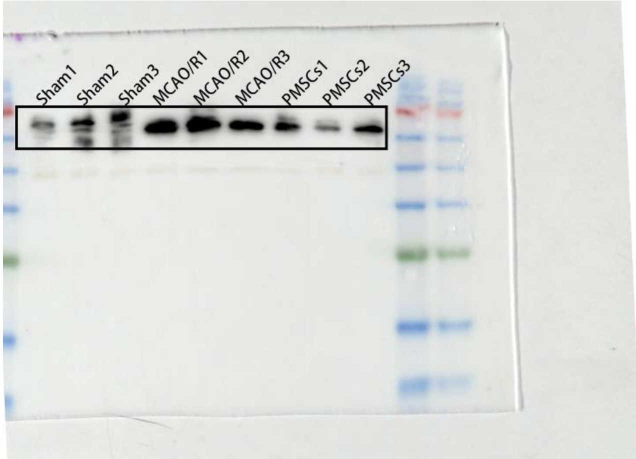

uncropped blot image of TBR2 in Fig.4F

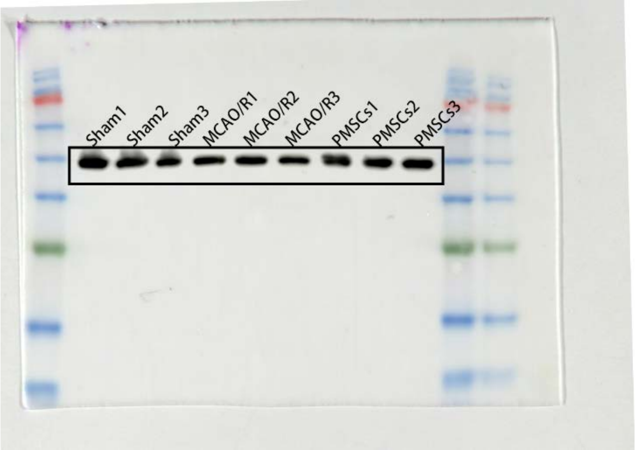

uncropped blot image of β-actin in Fig.4F-relative to TBRII

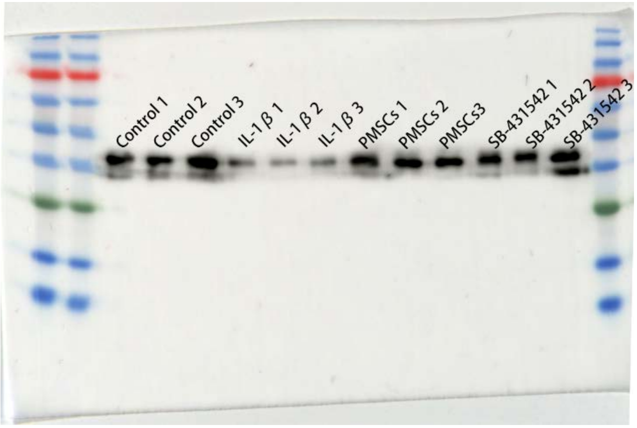

uncropped blot image of Arg-1 in Fig.5G

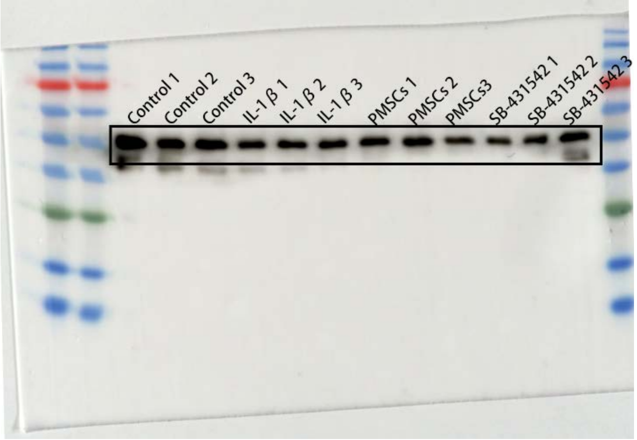

uncropped blot image of beta-actin in Fig.5G-relative to Arg1

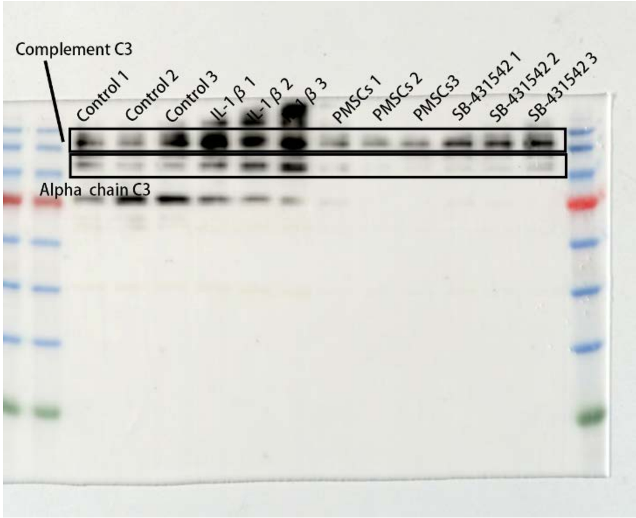

uncropped blot image of C3 in Fig.5G

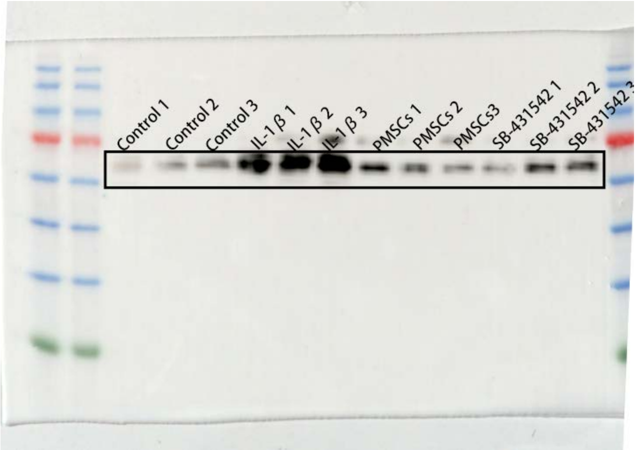

uncropped blot image of TβR2 in Fig.6A

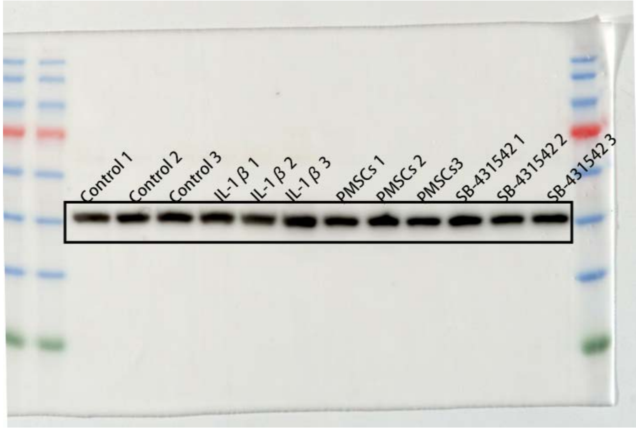

uncropped blot image of TβR2 in Fig.6A

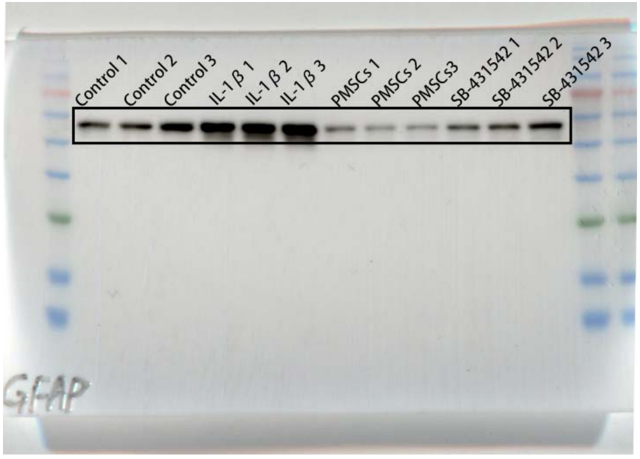

uncropped blot image of GFAP in Fig.5G

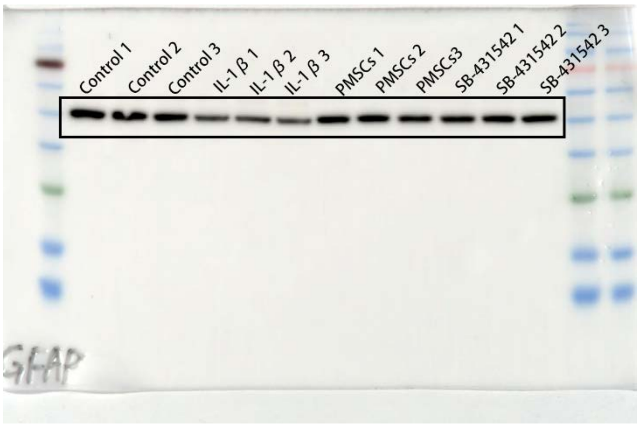

uncropped blot image of beta-actin in Fig.5G-relative to GFAP

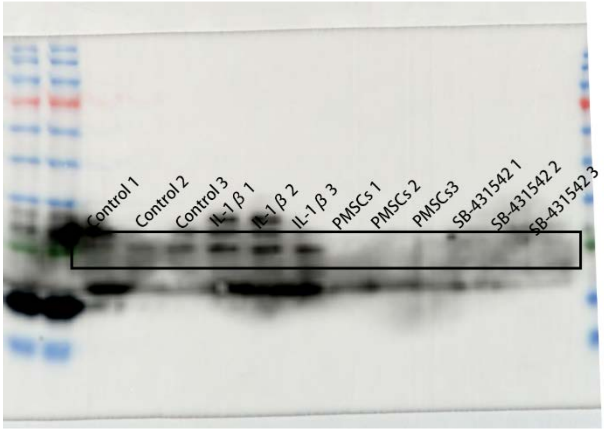

uncropped blot image of IL-6 in Fig.5G

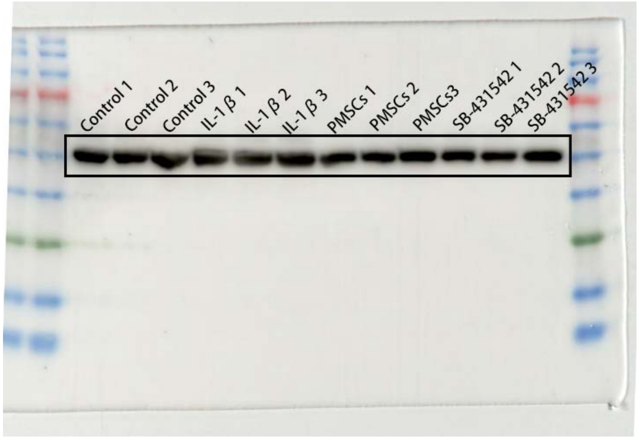

uncropped blot image of beta-actin in Fig.5G-relative to IL-6

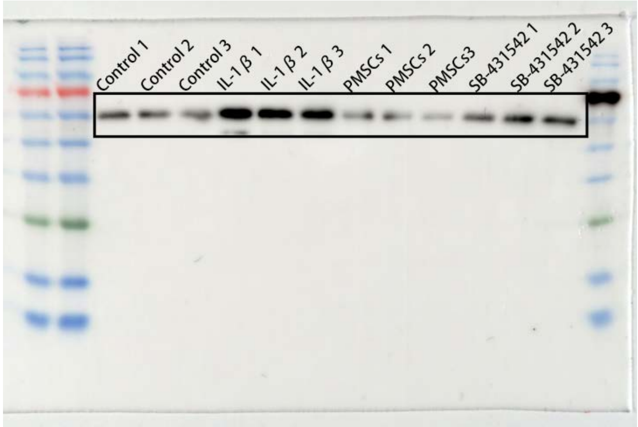

uncropped blot image of p-smad2-3 in Fig.6A

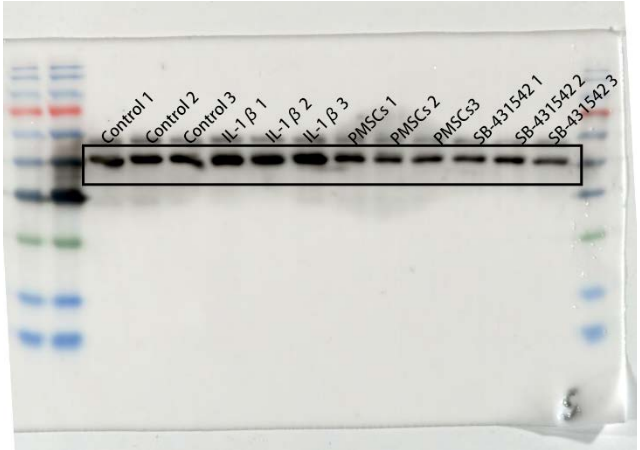

uncropped blot image of beta-actin in Fig.6A-relative to psmad2,3

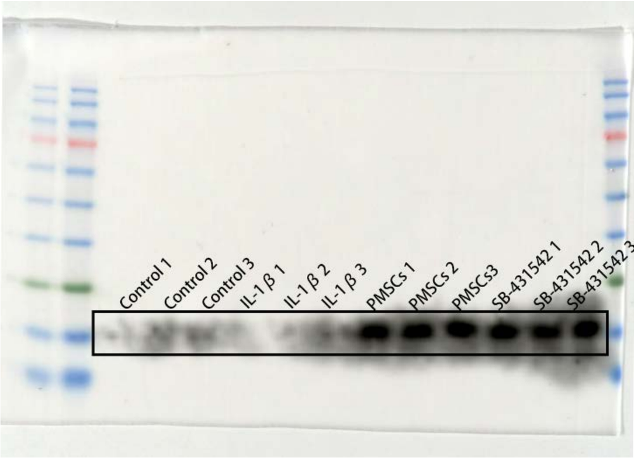

uncropped blot image of S100A10 in Fig.5G

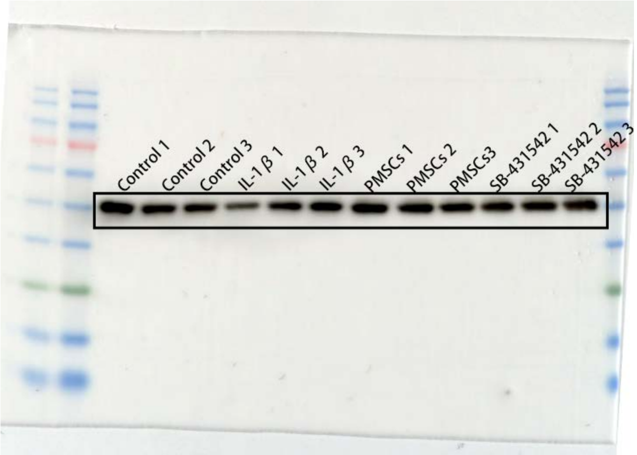

uncropped blot image of beta-actin in Fig.5G-relative to S100A10

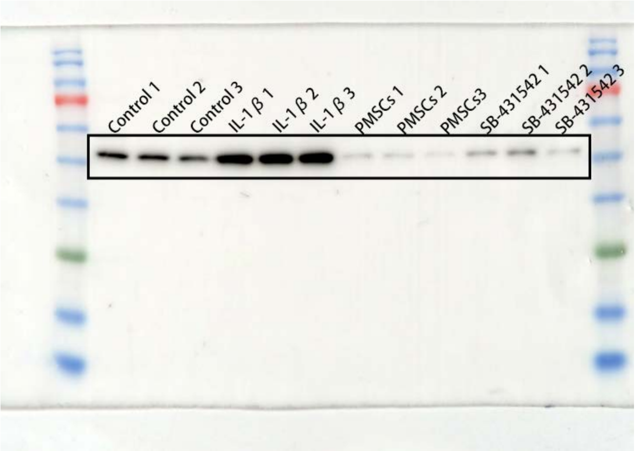

uncropped blot image of TGF-beta1 in Fig.6A

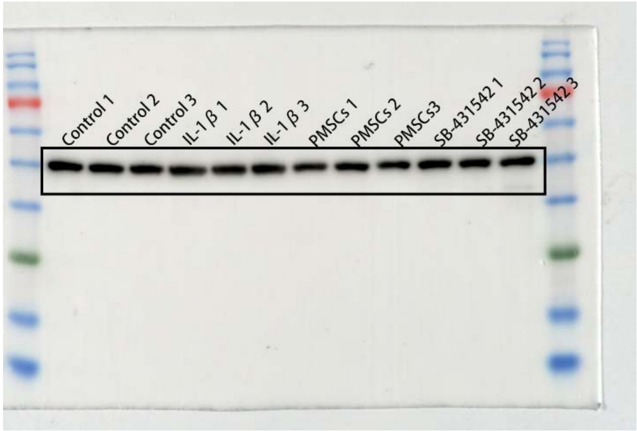

uncropped blot image of beta-actin in Fig.6A relative to TGFB1

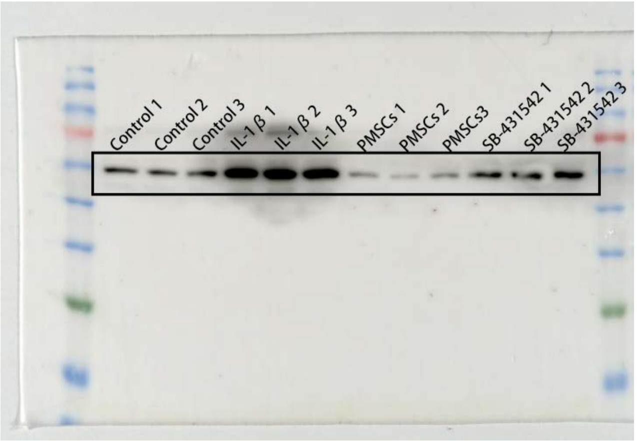

uncropped blot image of TβR1 in Fig.6A

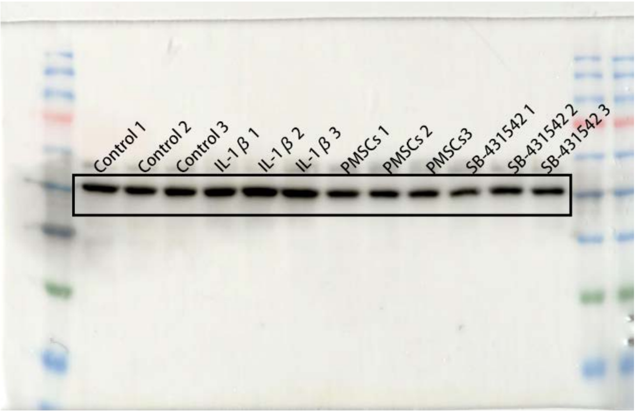

uncropped blot image of beta-actin in Fig.6A-relative to TBRI
